# Influence mechanism of structure on shear mechanical deformation characteristics of loess-steel interface

**DOI:** 10.1371/journal.pone.0263676

**Published:** 2022-02-07

**Authors:** Ya-zhi Wei, Zhi-hua Yao, Xiao-lei Chong, Jian-hua Zhang, Jun Zhang

**Affiliations:** 1 College of Aeronautical Engineering, Air Force Engineering University, Xi’an, China; 2 China Southwest Geotechnical Investigation & Design Institute Co., Ltd., Chengdu, China; University of Sharjah, UNITED ARAB EMIRATES

## Abstract

The mechanical properties of loess-steel interface are of great significance for understanding the residual strength and deformation of loess. However, the undisturbed loess has significant structural properties, while the remolded loess has weak structural properties. There are few reports on the mechanical properties of loess-steel interface from the structural point of view. This paper focused on the ring shear test between undisturbed loess as well as its remolded loess and steel interface under the same physical mechanics and test conditions (water content, shear rate and vertical pressure), and explored the influence mechanism of structure on the mechanical deformation characteristics of steel-loess interface. The results show that the shear rate has little effect on the residual strength of the undisturbed and remolded loess-steel interface. However, the water content has a significant influence on the residual strength of the loess-steel interface, moreover, the residual internal friction angle is the dominant factor supporting the residual strength of the loess-steel interface. In general, the residual strength of the undisturbed loess-steel interface is greater than that of the remolded loess specimen (for example, the maximum percentage of residual strength difference between undisturbed and remolded loess specimens under the same moisture content is 6.8%), which is because that compared with the mosaic arrangement structure of the remolded loess, the overhead arrangement structure of the undisturbed loess skeleton particles makes the loess particles on the loess-steel interface re-adjust the arrangement direction earlier and reach a stable speed relatively faster. The loess particles with angular angles in the undisturbed loess make the residual internal friction between the particles greater than the smoother particles of the remolded loess (for example, the maximum percentage of residual cohesion difference between undisturbed and remolded loess specimens under the same vertical pressure is 4.29%), and the intact cement between undisturbed loess particles brings stronger cohesion than the remolded loess particles with destroyed cement (for example, the maximum difference percentage of residual cohesion between undisturbed and remolded soil specimens under the same vertical pressure is 33.80%). The test results provide experimental basis for further revealing the influence mechanism of structure, and parameter basis for similar engineering construction.

## Introduction

Approximately 640,000 square kilometers of loess are distributed in China, which is the most representative regional special soil in China. With the in-depth development of the Belt and Road Initiative and the implementation of the new round of the Western Development Strategy, the construction projects in the loess area are increasing, which results in more interface engineering problems between loess and structures. For example, the pressing and drawing of pile foundation as well as the drawing of anchor rope (rod) are typical interface mechanical behaviors. It is known that the undisturbed loess has natural structural characteristics which keep the loess slope upright, forming special geomorphologic phenomena, such as soil bridges and soil forests. Therefore, the study of the impact of structure on the interface mechanical properties between loess and structure has an important application value. Mitchell [1] believed that the structure of soil was based on the arrangement and cementation between particles. Wu [2], Tan [3] studied the macroscopic mechanical characteristics of loess through triaxial test and Li [4], Wang [5] explained the microstructure characteristics of loess specimens before and after triaxial test. Jing [6], Xie [7] studied the interfacial shear behavior between loess and structure from the macro and micro perspectives. Ni [8], Xu [9] and Ge M [10] compared the changes of loess microstructure before and after permeability test by SEM. Jwa [11], Jia [12] analyzed the changes of loess microstructure in tensile and infinite lateral compression tests, respectively. Above results suggested that the structure had an important influence on the mechanical deformation properties of loess [13, 14]. The structure of undisturbed loess has a great influence on its mechanical characteristics in the process of extrusion and shear. However, after the destruction of undisturbed soil, its mechanical characteristics transit to those of remolded soil, that is, the structure is gradually lost. It is generally believed that the undisturbed loess has a strong structure, while the remolded loess has a weak structure due to the disappearance of particle bonding and cementation. Therefore, the structural reduction or disappearance of the undisturbed loess is also a manifestation of the transition from the mechanical properties of the undisturbed loess to the remolded loess soil. During the construction of pile foundation and the exertion of bearing capacity, the structure of undisturbed soil around pile will affect the mechanical effect of pile-soil interface. Therefore, it is necessary to study the influence of structure on the mechanical characteristics of soil-steel interface.

Most of the soil failures belong to shear failure, and the shear strength of soil specimen is one of the important parameters to study the mechanical properties of soil. Scholars both at home and abroad have carried out many studies on the shear strength of soil from the perspective of soil structure. HadžiNiković and Gordana D [15] analyzed the influence of natural soil structure on unsaturated shear strength through direct shear test. R-V. Matalucci [16–18] combined with triaxial tests with the structure model using Vixburg loess as granular soil material to explain that the structure has a significant impact on the ultimate strength. Li [19], Deng [20], Wang [5], Wei [21], Samoilych [22], Ng C.W.W. [23], Sadeghi [24], Ding [25], Nan [26], et al. analyzed the influence of microstructure and structural changes on the strength and deformation characteristics of soil. Li [27] found that there was a large difference between undisturbed tensile strength and disturbed tensile strength of loess, indicating the importance of its structure. Wen [28] studied the influence of structure on the shear properties of unsaturated loess through triaxial shear test. Leroueil [29] believed that there was a fundamental difference in terms of mechanical properties between the original state and the remodeling due to structural influence. However, in the actual construction of a large number of projects (such as pile foundation, anchor cable, anchor rod, etc.), loess often experiences shear with large displacement. Hvorslev [30] pointed out that the relatively good method for measuring the residual strength of soil was torsional shear. Therefore, although previous studies have discussed the influence of structure on the shear strength characteristics of unsaturated loess from both micro and macro perspectives, the commonly used shear strength test methods, such as direct shear test and triaxial test to understand the shear characteristics of loess under large deformation are inadequate. Many studies have shown that the ring shear apparatus can not only measure the residual strength by torsional shear, but also realize the study of large deformation shear characteristics between soil and structure [31–34]. Combined with the actual construction situation, the ring shear apparatus is selected to study the influence of residual strength from the structural point of view.

Currently, scholars both at home and abroad have done a lot of analyses and researches on the residual strength of soil by using ring shear apparatus. Wang [35], Kimura [36], Wang [37], Chen [38], Lian [39], Chen [40], Hu [41] and Scaringi [42] analyzed the influence rule of shear rate on residual shear strength of soil. Lian [43] et al. analyzed the influence of moisture content on stress-displacement relationship and residual strength parameters of loess. Xu [44] studied the influence of different normal stress on different rock-soil interface through ring shear test, and found that the peak shear strength and residual strength between different interfaces are different. Wang [45] found that polypropylene fiber can significantly improve the peak strength and residual strength of loess by ring shear test. Yuan [46] studied the shear characteristics of loess and paleosol under large deformation conditions by using ring shear test. Zou [47] et al. proposed the shear constitutive model of shear stress-displacement relationship of sliding zone soil based on the shear deformation failure characteristics and statistical damage theory of sliding zone soil ring shear test. The above studies discussed the residual strength of loess through ring shear test from different perspectives. However, the studies based on the analysis and comparison of the shear strength of unsaturated undisturbed and remolded loess from the structural point of view through the interface shear test are rarely mentioned.

It is known that there is a big difference in terms of mechanical properties between undisturbed loess and remolded loess. Therefore, it is of great significance to analyze the influence of structure on the mechanical properties of loess interface through ring shear test. In the context of pile foundation design and construction, the unsaturated undisturbed and remolded loess specimens were subjected to ring shear tests under different shear rates, water contents and vertical pressures. Combined with the SEM scanning and CT scanning test results of undisturbed loess and remolded loess, the influence of structural differences between undisturbed loess and remolded loess on the residual strength and deformation law of loess-steel interface was studied and the shear strength of loess was analyzed from a new point of view. It provides an important experimental basis for further revealing the influence of structure on the mechanical deformation characteristics of loess-steel interface, and also an important scientific basis for denying pile foundation, anchor cable (rod) and other engineering constructions in the loess area of central and western China. The main structure of this paper is organized as follows. In the second section, the test materials, instruments and schemes are described in detail. In the third section, the stress-strain relationship and residual strength parameters of the loess-steel interface under the influence of three basic factors are expounded. And in the fourth section, the difference in terms of the microstructure characteristics of undisturbed loess and remolded loess as well as the influence mechanism of structural differences on the residual strength characteristics of loess-steel interface under the influence of three factors are discussed.

## Materials and methods

### Specimen preparation

**Fig 1:**
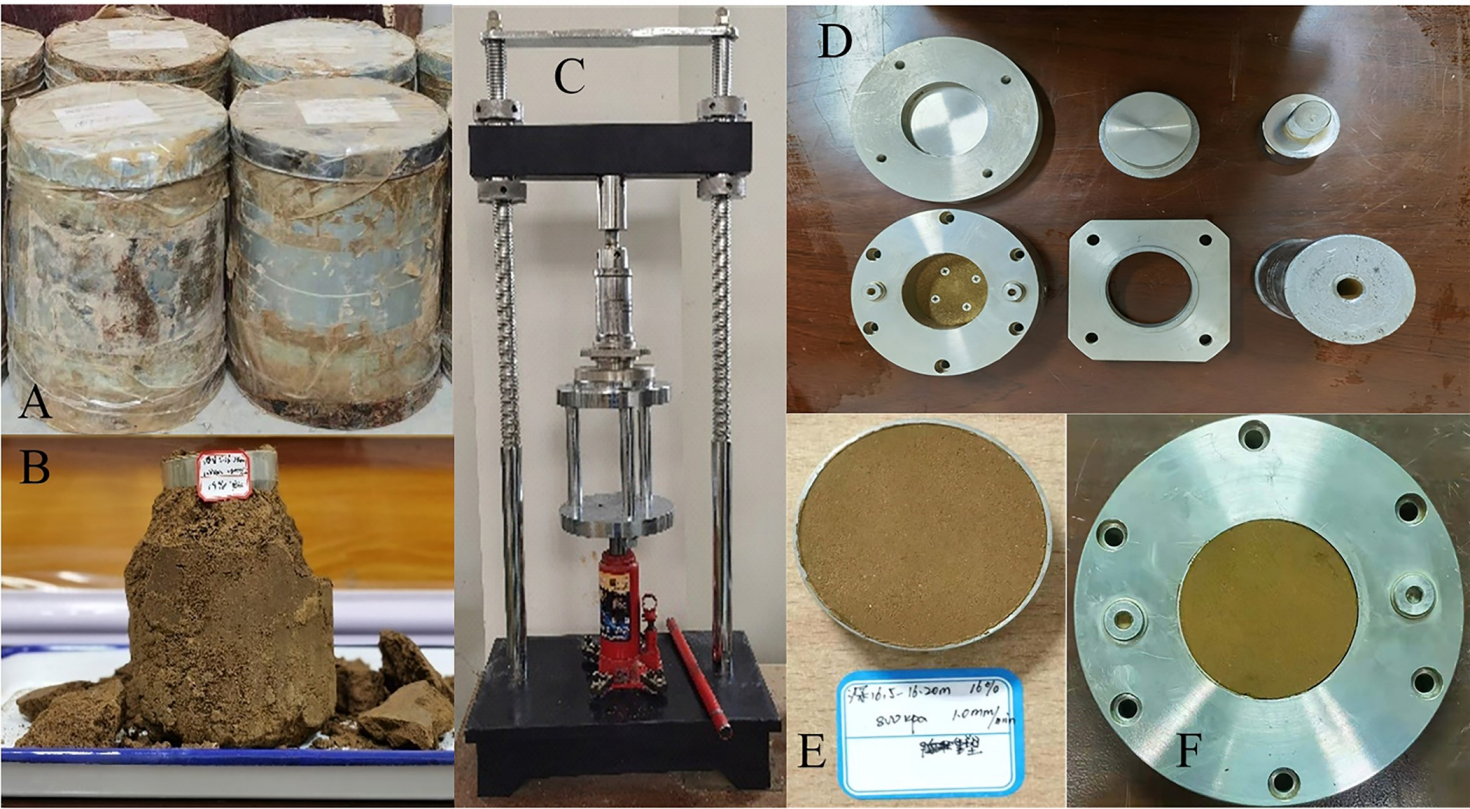
Sampling equipments and methods for undisturbed and remolded loess. **(A)** Undisturbed loess specimen sealed in iron cylinder. **(B)** Preparation of undisturbed loess cutting ring specimen. **(C)** Remodeling specimen preparation equipment. **(D)** Reshaping specimen preparation tool. **(E)** Remodeling cutting ring specimen. **(F)** The remolded cutting ring specimen into the shear box.

The specimens were taken from Jingwei New Town, Xi ’ an, Shaanxi. Exploratory wells were excavated manually on the site, and a number of undisturbed loess specimens in the diameter of 12 cm and height of 15 cm were obtained per linear meter. To prevent water evaporation, the specimens were sealed with iron cylinder and transported back to the laboratory (as shown in (Fig 1A)). The specific gravity of loess particles used in the test is 2.71, and the other physical indexes are listed in Table 1. Furthermore, the chemical composition of the tested loess is as follows: quartz: 49.8%, plagioclase: 15.6%, potassium feldspar: 1.8%, calcite: 11.9%, dolomite: 1.6%, illite: 8.6%, chlorite: 8.5%, pyrite: 0.7%, amphibole: 1.5% [48].

**Table 1:** Initial physical index of undisturbed loess specimen.

Buried depth of specimen	Initial water content	Dry density	Void ratio	Saturation	Liquid limit	Plastic limit
(m)	*w* (%)	*ρ*_*d*_ (g/cm3)	*e*	*S*_*r*_(%)	*w*_*L*_ (%)	*w*_*P*_(%)
6.5	16.25	1.35	1.01	43.60	32.70	16.94

**Fig 2:**
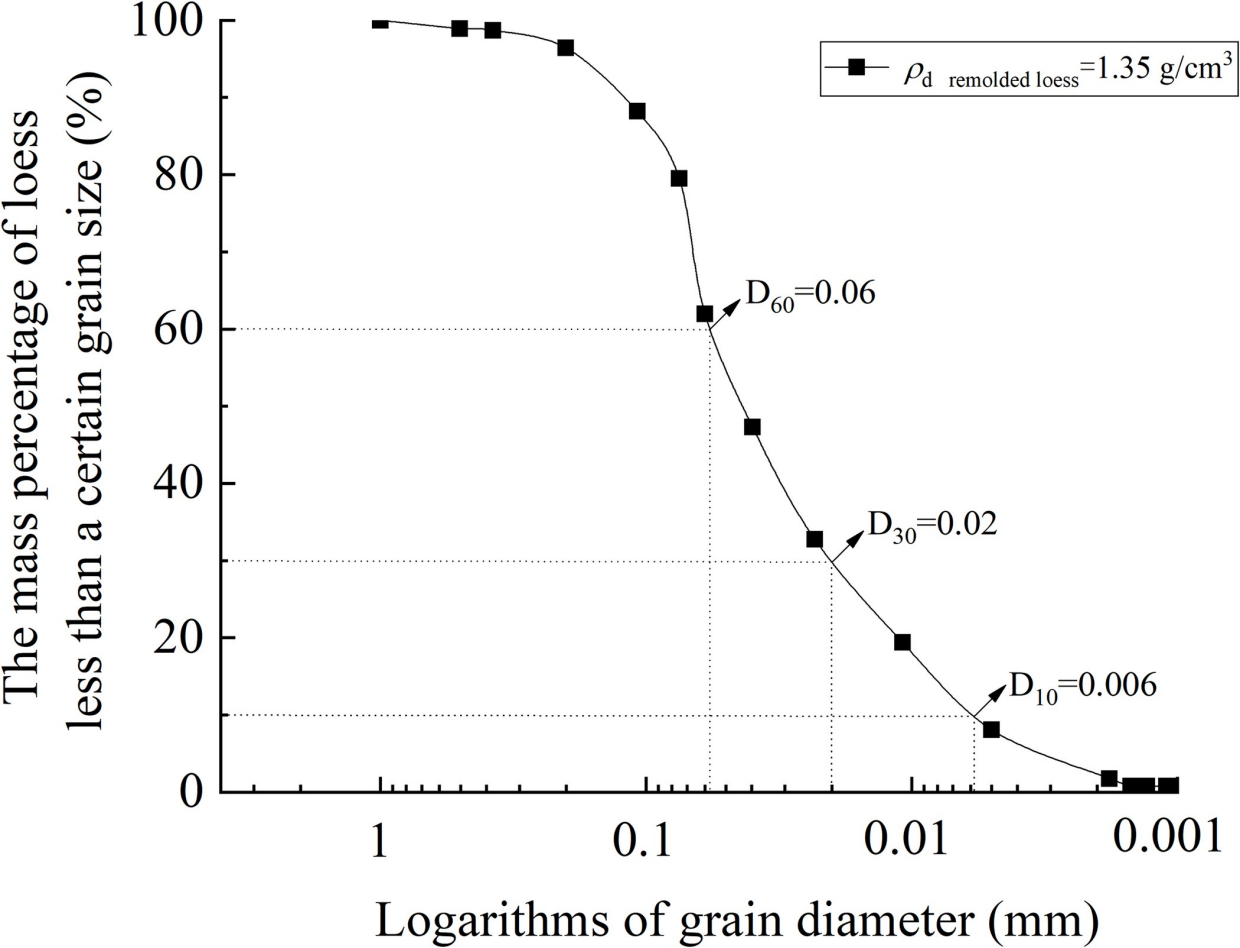
Grain grading curve of remolded loess specimen.

The undisturbed loess interface shear specimen is pressed into the loess vertically by the ring knife until the ring knife cylinder is filled with loess specimen. Then, the loess specimen around the ring knife are cut with the loess cutting knife, and the ring knife filled with loess is taken out. The excess loess at both ends of the ring knife is cut off, and the loess outside the ring knife is wiped (as shown in (Fig 1B)) to obtain an undisturbed loess ring knife loess specimen in the diameter of 61.8 mm and the height of 20 mm. The remaining loess specimen of the undisturbed specimen is used for the preparation of the remolded specimen, which is quickly crushed through the1 mm sieve. The dry density and water content of the remolded loess specimen are controlled to be the same as those of the undisturbed loess specimen, and the remolded loess specimen is compacted by the remolded loess specimen preparation equipment (as shown in (Fig 1C and 1D)). The specimen size is the same as that of the undisturbed loess specimen (as shown in (Fig 1E and 1F)). In addition, the particle size distribution curve of the remolded loess specimen is shown in Fig 2.

The natural water content of the undisturbed specimen is 12.69%. Using the 5mL syringe, water is dropped on the undisturbed specimen in several lots, and the interval between each drop is 2–3 hours. Referring to the test results of loess specimens with similar dry density in the existing literature [49, 50], the water content is adjusted to 16%, 19% and 22%, respectively according to the test scheme. Put the prepared specimen into the moisturizing cylinder, and to make the moisture evenly distributed, the specimen should be placed for not less than 72 hours, and turned every 12 hours. In the meantime, to avoid the evaporation of the moisture of the specimen in the humidifier, it is necessary to weigh the specimen and make sure the mass of the specimen is equal to that of the specimen after the addition of water when turning the specimen. In the case that mass is slightly reduced, water must be dripped again to achieve the target water content. The water content of remolded loess is achieved mainly by drying the screened loess, and accurately preparing the loess with the target water content. The preparation process of loess water content can be based on the specification [51].

### Test apparatus

**Fig 3:**
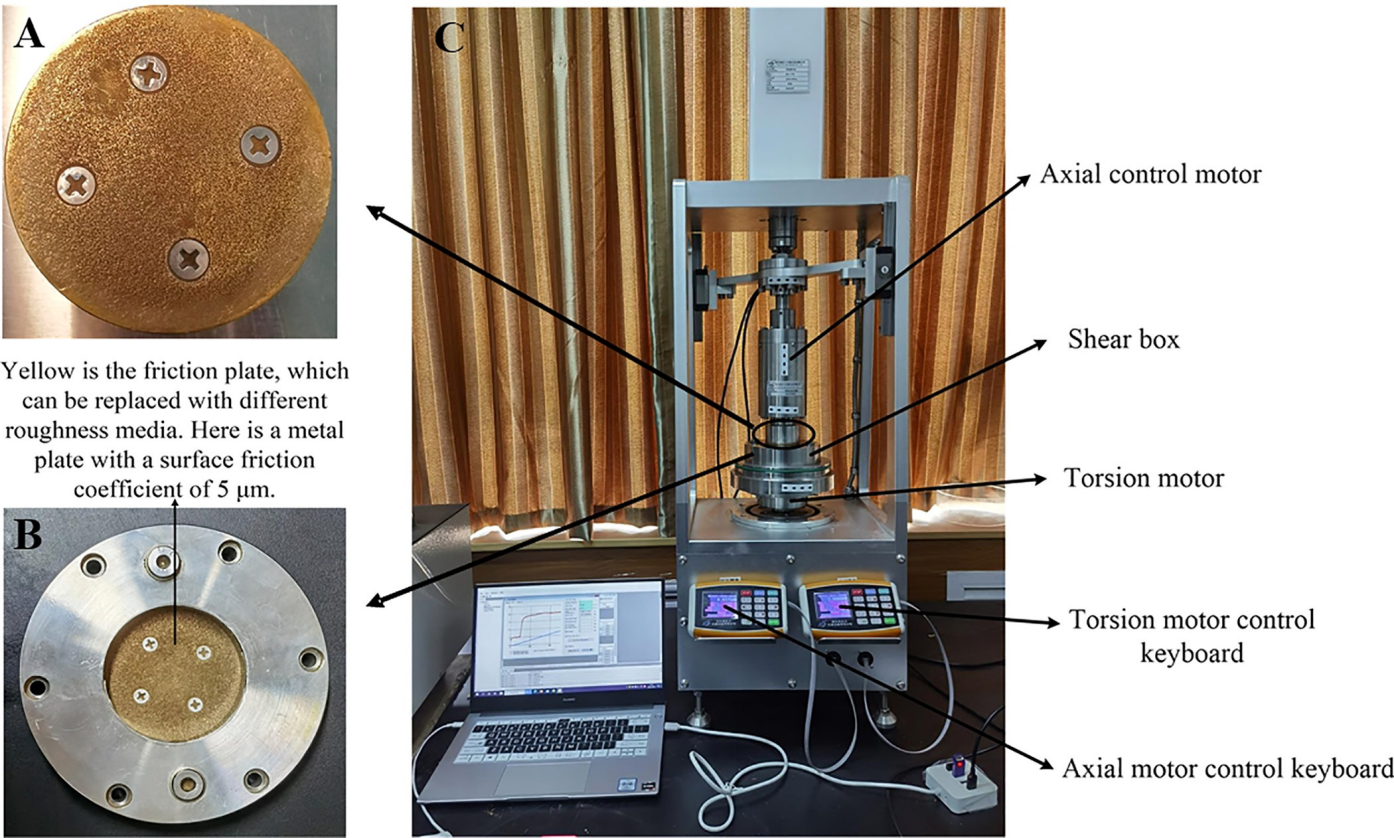
Interface shear test instrument & shear box. **(A)** Shear head. **(B)** Shear box. **(C)** Interface shear test instrument.

This test adopts KTL-TTS interface shear apparatus, which includes an axial load system and a torsional load system. The built-in composite sensor can be used to measure the axial load and the torque loaded on the specimen. The axial part is driven by the stepper motor at the top of the equipment, and the axial force is closed-loop controlled by the feedback of the sensor. The torsional part is driven by the motor at the bottom of the equipment, and the torque is closed-loop controlled by the feedback of the sensor. The maximum shear velocity of the apparatus is 125°/ min, and the maximum vertical pressure can reach 1200 kPa, besides, and the vertical displacement accuracy can be as high as 0.0001 mm. The shear box used in the test is 61.8 mm in diameter and 20 mm in depth, which is in the same size as the standard ring cutter. The details of the instrument and shear box are shown in Fig 3A–3C.

Compared with the conventional direct shear apparatus, this apparatus can ensure that the shear surface is constant and the force is uniform during the shear process. Moreover, the base can rotate without limit of angle, that is, the shear angle is arbitrary. The key data in the ring shear test is shear torque, which is automatically collected by the computer during the shear process. The calculation formula is expressed as follows:

1) When the apparatus rotates, Torque *M* (*N*⋅*mm*) is:

$$M=\int_{0}^{R}\tau_{n}\times 2\pi R^{2}dR$$ (1)

where, *τ*_*n*_ refers to the average shear stress (kPa), and *R* represents the radius of the specimen (mm). Besides, the radius of the specimen used in this test is 30.9 mm.

The average shear stress on the rotating surface can be obtained as:

$$\tau_{n}=\frac{3M}{2\pi R^{3}}$$ (2)

2) The vertical pressure on the rotating surface is:

$$\sigma_{n}=\frac{W}{\pi R^{2}}$$ (3)

where, *W* refers to the vertical load (*N*).

Under the constant vertical pressure, the specimen will have vertical displacement in the shear process. The vertical displacement is divided by the height of the specimen after consolidation to obtain the vertical strain in the shear process of the specimen.

3) The average displacement on the rotating surface is related to the rotational speed and angular displacement of the equipment. The calculation formula is as follows:

$$S=\pi D\times v\times t=\frac{D}{2}\theta$$ (4)

where, *v* refers to the speed (° / min.), *t* represents the time (min.), *θ* denotes the angular displacement (°), and the *D* stands for the average diameter of the specimen (mm).

### Test method

To make the test results consistent with the actual situation, the vertical pressure corresponding to the ring shear test should be applied to the loess specimen before the shear test. The consolidation stability standard is that the vertical deformation within 1 h does not exceed 0.01 mm [52]. To explore the influence of related factors on the peak strength and residual strength of undisturbed and remolded loess specimens, the interface shear test is carried out using three influence factors, including shear rate, water content and vertical pressure as variables. The vertical pressure is selected as 100 kPa, 200 kPa, 400 kPa, 800 kPa and 1200 kPa, and the shear rate is selected as 0.1 mm / min, 1.0 mm / min and 5.0 mm / min (0.253° / min, 2.529° / min and 12.645° / min). Besides, the water content is selected 16%, 19% and 22%, respectively.

**Fig 4:**
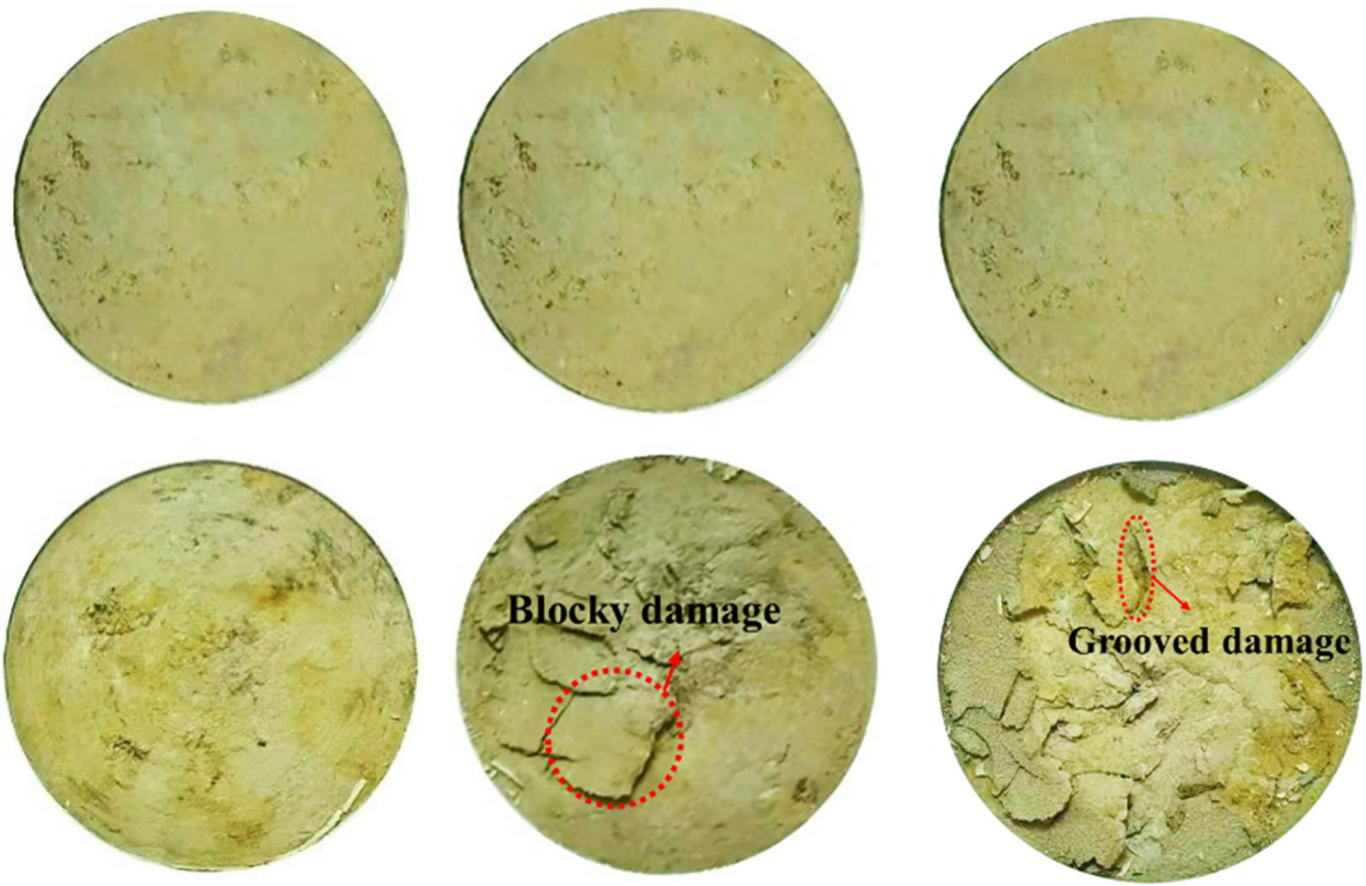
Comparison of the shear surface morphology of 1^#^ - 3^#^ undisturbed loess specimen before and after the test. **(A**) 1^#^ *v* = 0.1 mm / min. **(B)** 2^#^ *v* = 1.0 mm / min. **(C)** 3^#^ *v* = 5.0 mm / min.

Before the test, the compaction device should be used to apply a small pressure to the consolidated ring knife loess specimen to press the loess specimen from the ring knife into the shear box, as shown in Fig 4. In this paper, a total of 36 tests is carried out for the undisturbed and remolded loess specimen with 18 tests for each of them. The number 1^#^ - 18^#^ refers to the undisturbed loess specimen, and 19^#^-36^#^ represents the remolded loess specimen. The influence of shear rate is compared using the specimens of 1^#^ - 3^#^ and 19^#^ - 21^#^, the water content is 19% and the vertical pressure is 400 kPa; the specimens of 4^#^ - 6^#^ and 22^#^ - 24^#^ are used for the comparison of the influence of water content, the shear rate is 1.0 mm / min and vertical pressure is 800 kPa; the influence of vertical pressure is compared using the specimens of 4^#^ - 18^#^ and 2^#^ - 36^#^. The shear rate is 1.0 mm / min and the water content is 16%, 19% and 22% The specific test scheme is shown in Table 2.

**Table 2:** Test plan.

Specimen Nos.		Invariant factors		
Undisturbed loess	Remolded loess	*v* (mm/min)	*w (%)*	*σ* (kPa)
1^#^	19^#^	0.1	19	400
2^#^	20^#^	1.0		
3^#^	21^#^	5.0		
4^#^	22^#^	1.0	16	800
5^#^	23^#^		19	
6^#^	24^#^		22	
7^#^	25^#^	1.0	16	100
8^#^	26^#^			200
9^#^	27^#^			400
4^#^	22^#^			800
10^#^	28^#^			1200
11^#^	29^#^	1.0	19	100
12^#^	30^#^			200
13^#^	31^#^			400
5^#^	23^#^			800
14^#^	32^#^			1200
15^#^	33^#^	1.0	22	100
16^#^	34^#^			200
17^#^	35^#^			400
6^#^	24^#^			800
18^#^	36^#^			1200

### Test results and analysis

The strength of the specimen is basically stabilized at a fixed value, that is, on the shear stress–shear displacement curve of the specimen, when the shear stress exceeds the peak strength (the highest point of shear stress), it gradually decreases to a constant and maintains basically stable (namely residual strength). For the length of the shear displacement to set the value of residual strength, there is no clear stipulation at present. In this paper, the shear stress corresponding to the shear displacement of 60 mm is taken as the residual strength value. At this time, the residual strength change is small, and it can be considered as a stable value.

### Impact of different shear rates on deformation and strength characteristics of undisturbed and remolded loess specimens

**Fig 5:**
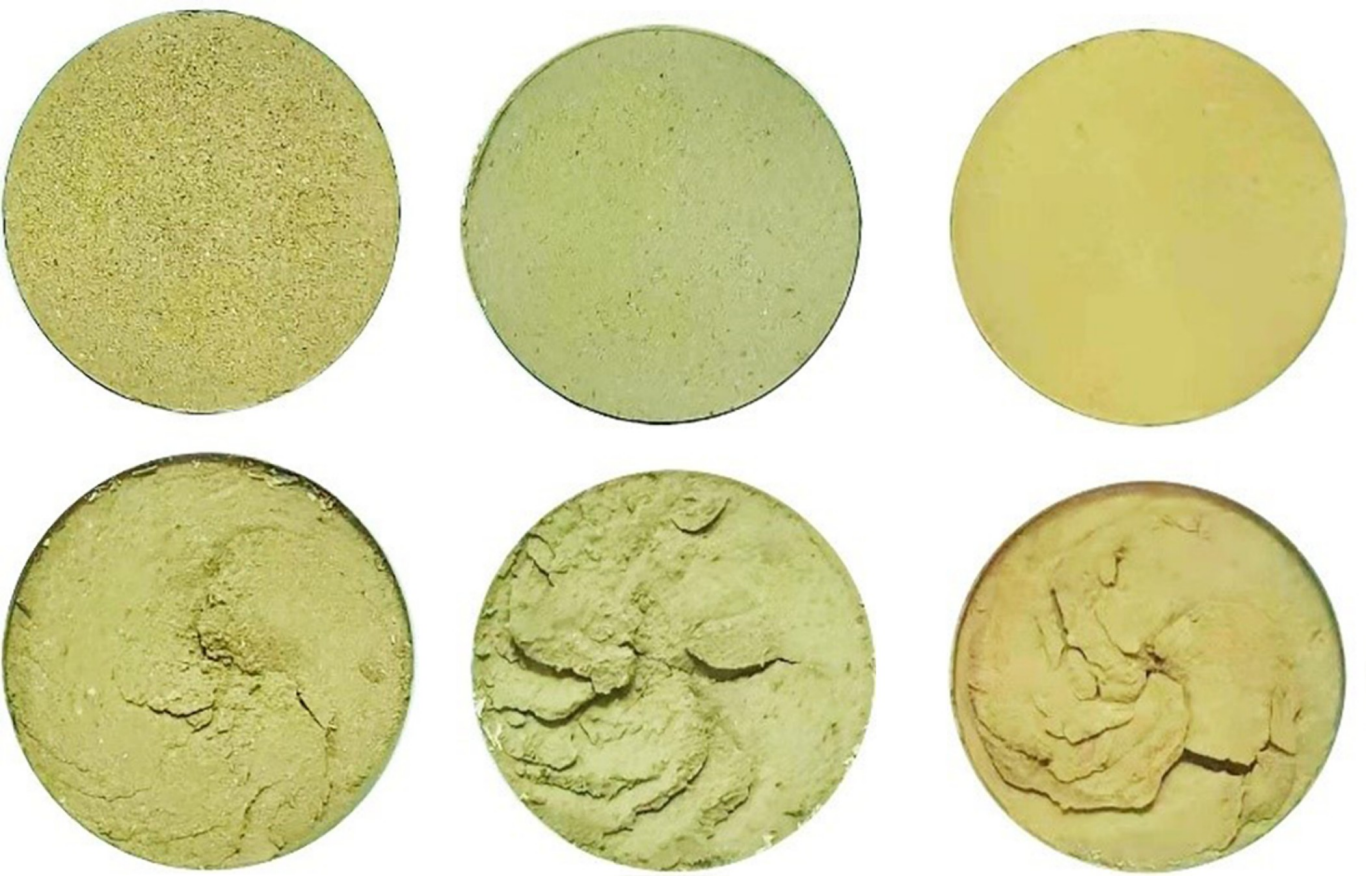
Comparison of the shear surface morphology of 19^#^ - 21^#^ remolded loess specimen before and after the test. **(A)** 19^#^ *v* = 0.1 mm / min. **(B)** 20^#^ *v* = 1.0 mm / min. **(C)** 21^#^ *v* = 5.0 mm / min.

**Fig 6:**
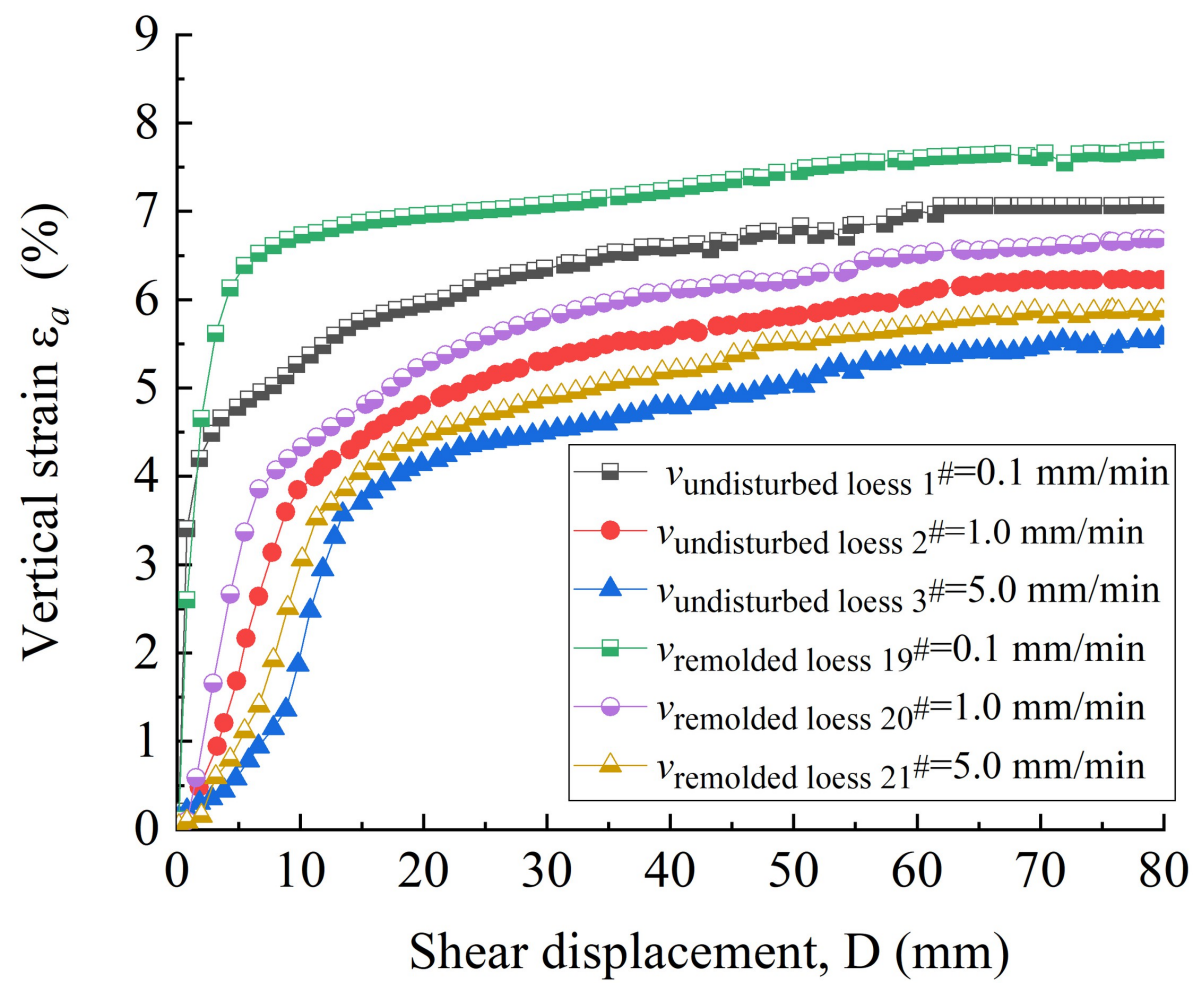
Vertical strain-shear displacement curve of 1^#^ - 3^#^ undisturbed and 19^#^ - 21^#^ remolded loess specimens under different shear rates.

The shear surface morphology of the undisturbed 1^#^-3^#^ and remolded 19^#^ - 21^#^ specimens before and after shearing is shown in Figs 4A–4C and 5A–5C, respectively. It can be seen that the variation of shear surface morphology of 1^#^ and 19^#^ loess specimens before and after shear at the shear rate of 0.1 mm / min is slightly small; under the shear rate of 2^#^ and 20^#^ at 1.0 mm/min and 3^#^ and 21^#^ at 5.0 mm / min, the change degree of shear surface morphology before and after shear increases with the increase of shear rate. This is because that the faster the shear rate, the deeper the loess is destroyed, and the thicker the shear band. In addition, the color of the loess specimen at the shear surface after the test is slightly deeper than that before the shear, which is caused by the fact that the pore water gradually accumulates to the shear surface as the shear displacement increases. At low shear rate, the friction plate rotates and squeezes the surface of the specimen, and the weakly bound water of the loess particles and their aggregates is discharged under the action of the overlying vertical pressure and shear force. The discharged water then forms a water film on the surface of the metal plate and the specimen. The water film plays a lubricating role between the metal plate and the specimen surface, which in turn affects the friction strength between the metal friction plate and the loess specimen. Under high shear rate, the water on the surface of the specimen is squeezed and discharged during shearing, which enhances the bonding effect between the steel interface and the loess particles. The steel friction plate is bonded with large loess aggregates, which increases the thickness of the shear band below the surface of the specimen. Besides, the groove and block failure structure characteristics appear. Fig 6 shows the variation curves of the vertical strain of 1^#^ - 3^#^ and 19^#^ - 21^#^ specimens with shear displacement. As can be seen from Fig 6, the shear failure of the specimen is a shear contraction process, that is, the height of the specimen decreases with the increase of shear displacement, and the change trend of undisturbed and remolded specimens under different shear rates is basically the same, while the change trend of undisturbed and remolded specimens under the same shear rate is higher. At the shear rate of 0.1 mm / min, when the shear displacement of 1^#^ and 19^#^ specimens is 60 mm, the corresponding vertical strains are roughly 4.9% and 5.6%; at the shear rate of 1.0 mm / min, when the shear displacement of 2^#^ and 20^#^ specimens is 60 mm, the corresponding vertical strains are roughly 5.9% and 6.5%; at the shear rate of 5.0 mm / min, the corresponding vertical strain of 3^#^ and 21^#^ specimens is roughly 6.9% and 7.6% when the shear displacement is 60 mm.

**Fig 7:**
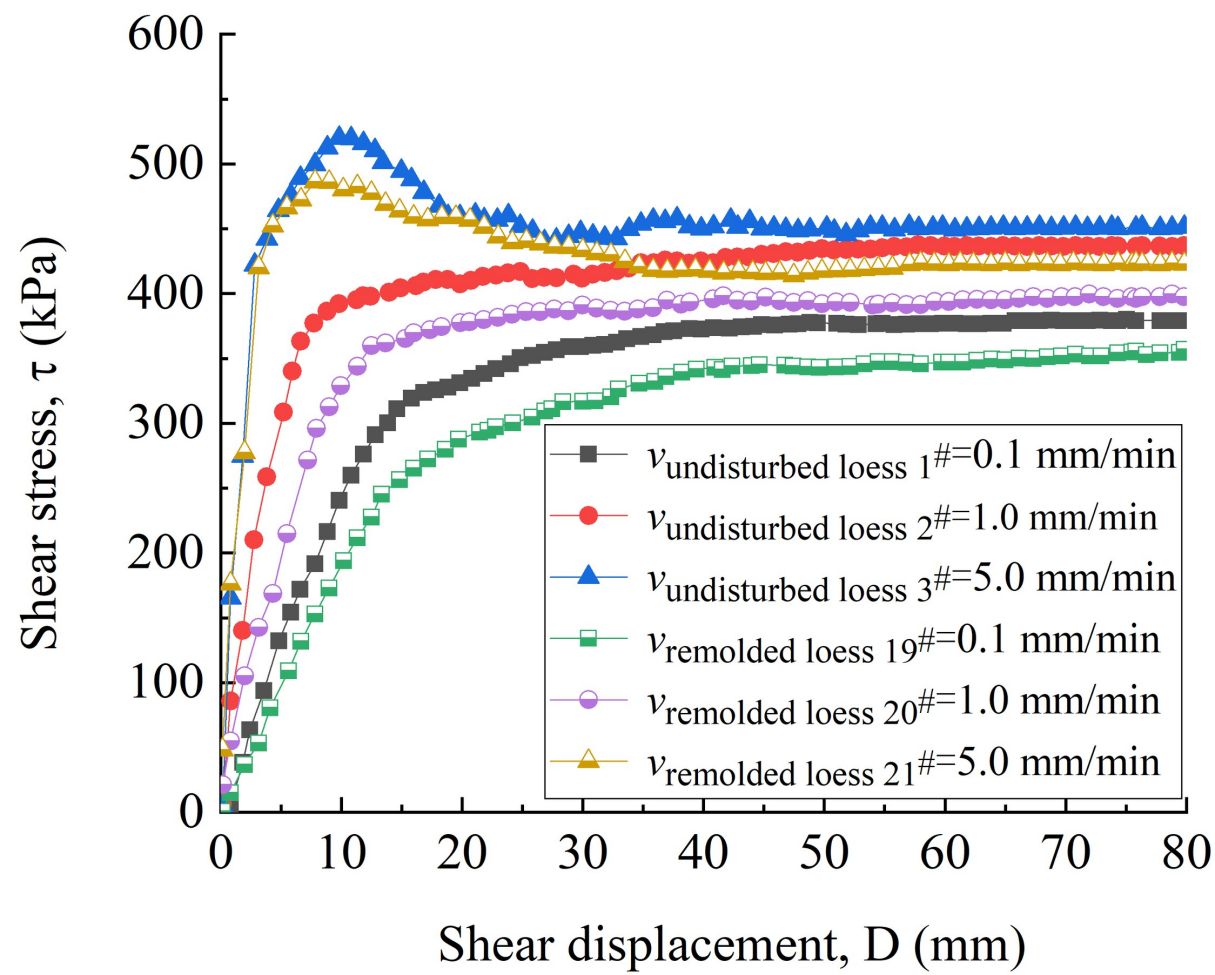
Shear stress-shear displacement curve of 1^#^ - 3^#^ undisturbed and 19^#^ - 21^#^ remolded loess specimens under different shear rates.

Fig 7 shows the variation curves of shear stress with shear displacement of undisturbed 1^#^ - 3^#^ and remolded 19^#^ - 21^#^ specimens. It can be seen from Fig 7 that under the shear rate of 0.1 mm / min, the shear stress–shear displacement curves of 1^#^ and 19^#^ loess specimens show a weak hardening type. At the shear rate of 1.0 mm / min, the shear stress-shear displacement curves of 2^#^ and 20^#^ loess specimens are strong hardening type. At the shear rate of 5.0 mm / min, the shear stress-shear displacement curves of 3^#^ and 21^#^ are soft type. The change of shear stress of loess specimens under three shear rates can be roughly divided into two stages. At the shear rate of 0.1 mm / min, the first stage of 1^#^ and 19^#^ loess specimens occurs at the stage of shear displacement of 0 ~ 30 mm and 0 ~ 40 mm, respectively. Then, the shear stress rapidly increases to a higher strength with the increase of shear displacement (about 400 kPa and 365 kPa, respectively). In this stage, with the occurrence of shear failure, the internal specimen begins to show tiny cracks, and the increase of shear displacement makes the cracks through and form a continuous shear surface; after entering the second stage, the shear displacement continues to increase. The shear stress of undisturbed and remolded loess specimens slowly changes within the range of 400 ~ 423 kPa and 365 ~ 388 kPa, respectively, and reaches a stable residual strength when the shear displacement is about 60 mm. At this stage, the loess particles on the internal shear surface of the loess specimen move and rearrange along the shear direction, and finally complete the directional arrangement to achieve the stability. At the shear rate of 1.0 mm / min, the first stage of 2^#^ and 20^#^ loess specimens occurs at the shear displacement of 0 ~ 42 mm and 0 ~ 45 mm, respectively, and rapidly increases to a higher strength (about 402 kPa and 380 kPa, respectively). When the shear displacement is about 60 mm, the stable residual strength is 436 kPa and 396 kPa, respectively. At the shear rate of 5.0 mm / min, the shear surface of 3^#^ and 21^#^ loess specimens forms the first stage shear surface at the shear displacement of 9 mm and 7 mm, respectively, reaching the peak strength (about 520 kPa and 486 kPa), and then the stable residual strength is 450 kPa and 417 kPa when the shear displacement is 60 mm. The comparison shows that the peak strength and residual strength of the undisturbed loess specimen at the same shear rate are slightly higher than those of the remolded loess specimen, and the shear displacement required for the remolded loess specimen to reach higher strength in the first stage is greater than that of the undisturbed loess specimen. Comparing the peak and residual strength of undisturbed loess and remolded loess specimens under different shear rates, it can be seen that the higher the shear rate, the greater the peak strength of the loess specimen, and the shear rate has little effect on the residual strength of the loess specimen.

**Fig 8:**
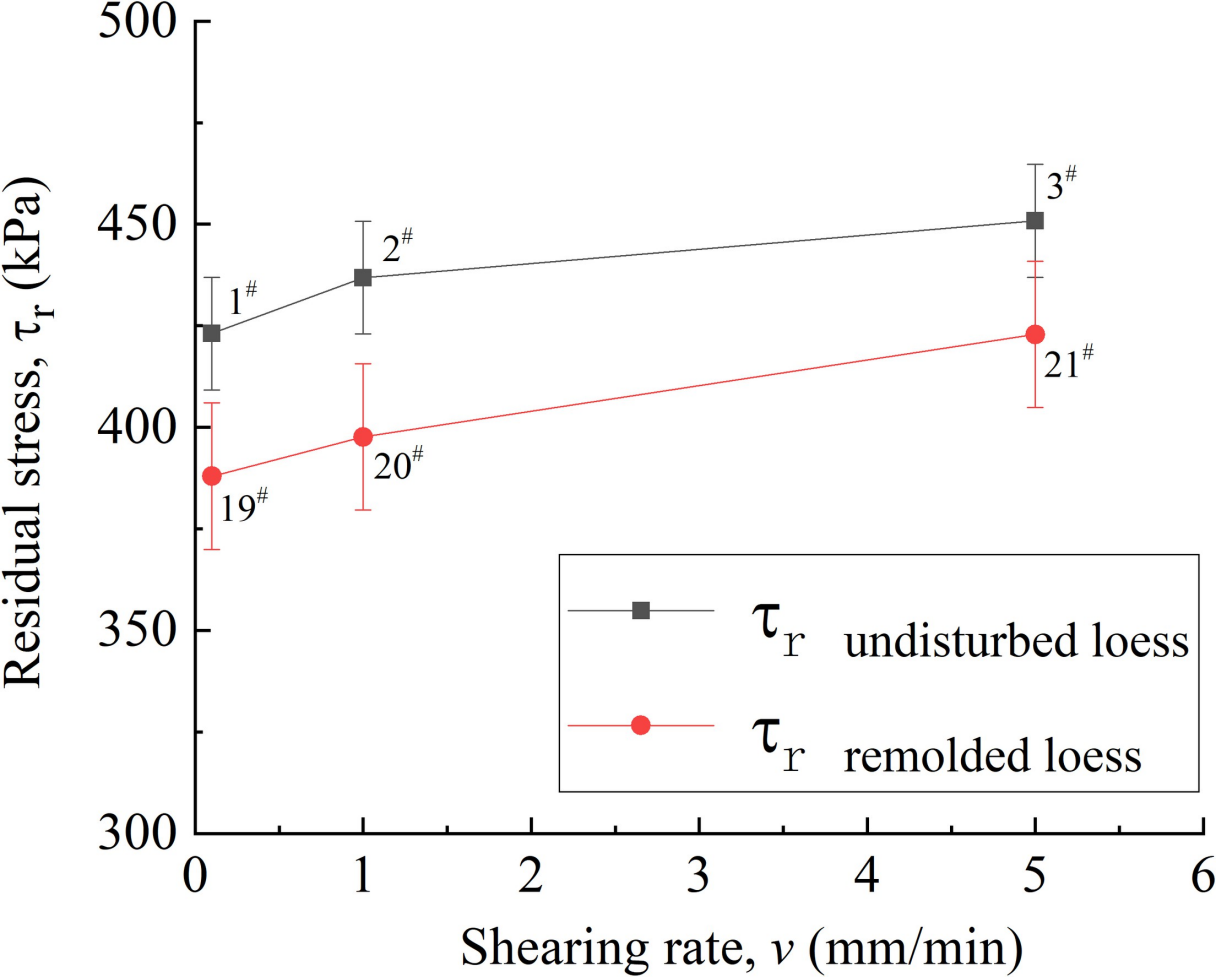
Residual strain-shearing rate curve of 1^#^ - 3^#^ undisturbed and 19^#^ - 21^#^ remolded loess specimens.

Fig 8 shows the relationship between shear rate and residual strength of undisturbed 1^#^ - 3^#^and remolded 19^#^ - 21^#^ specimens. It can be seen from Fig 8 that when the shear rate is 0.1 mm / min, 1.0 mm / min and 5.0 mm / min, the residual strength of 1^#^ - 3^#^ loess specimens is 423 kPa, 436 kPa and 450 kPa, respectively, and the strength difference range is 3.0% ~ 6.5%. The residual strengths of 19^#^ - 21^#^ loess specimens are 388 kPa, 396 kPa and 417kPa, respectively, and the strength difference range is in 2.0% ~ 7.5%. Therefore, under a certain shear rate, for other undisturbed and remolded loess specimens under the same conditions, the influence of shear rate on residual strength is small.

### Impact of different water content on deformation and strength characteristics of undisturbed and remolded loess specimens

**Fig 9:**
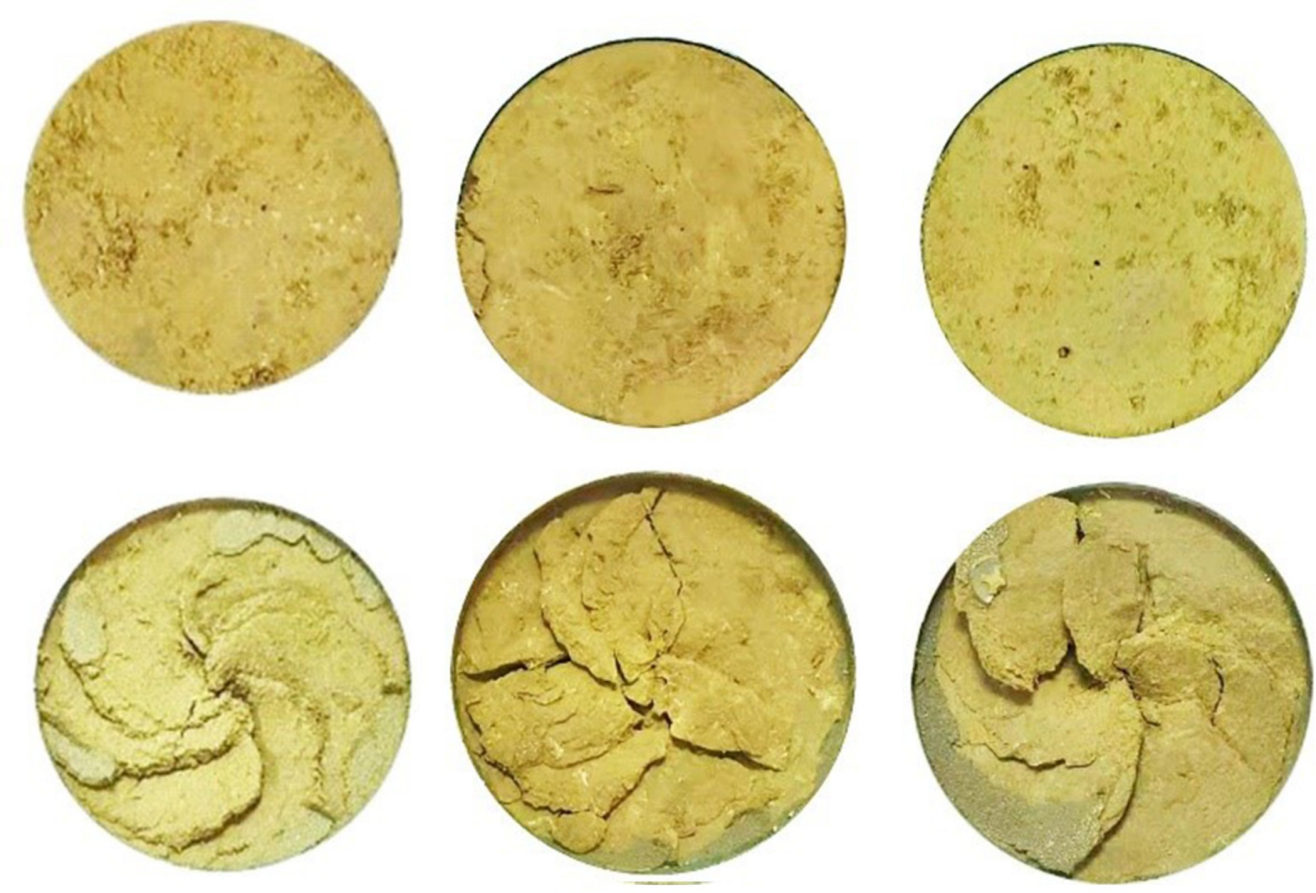
Comparison of the shear surface morphology of 4^#^ - 6^#^ undisturbed loess specimen before and after the test. **(A)** 4^#^ *w* = 16%. **(B)** 5^#^ *w* = 19%. **(C)** 6^#^ *w* = 22%.

**Fig 10:**
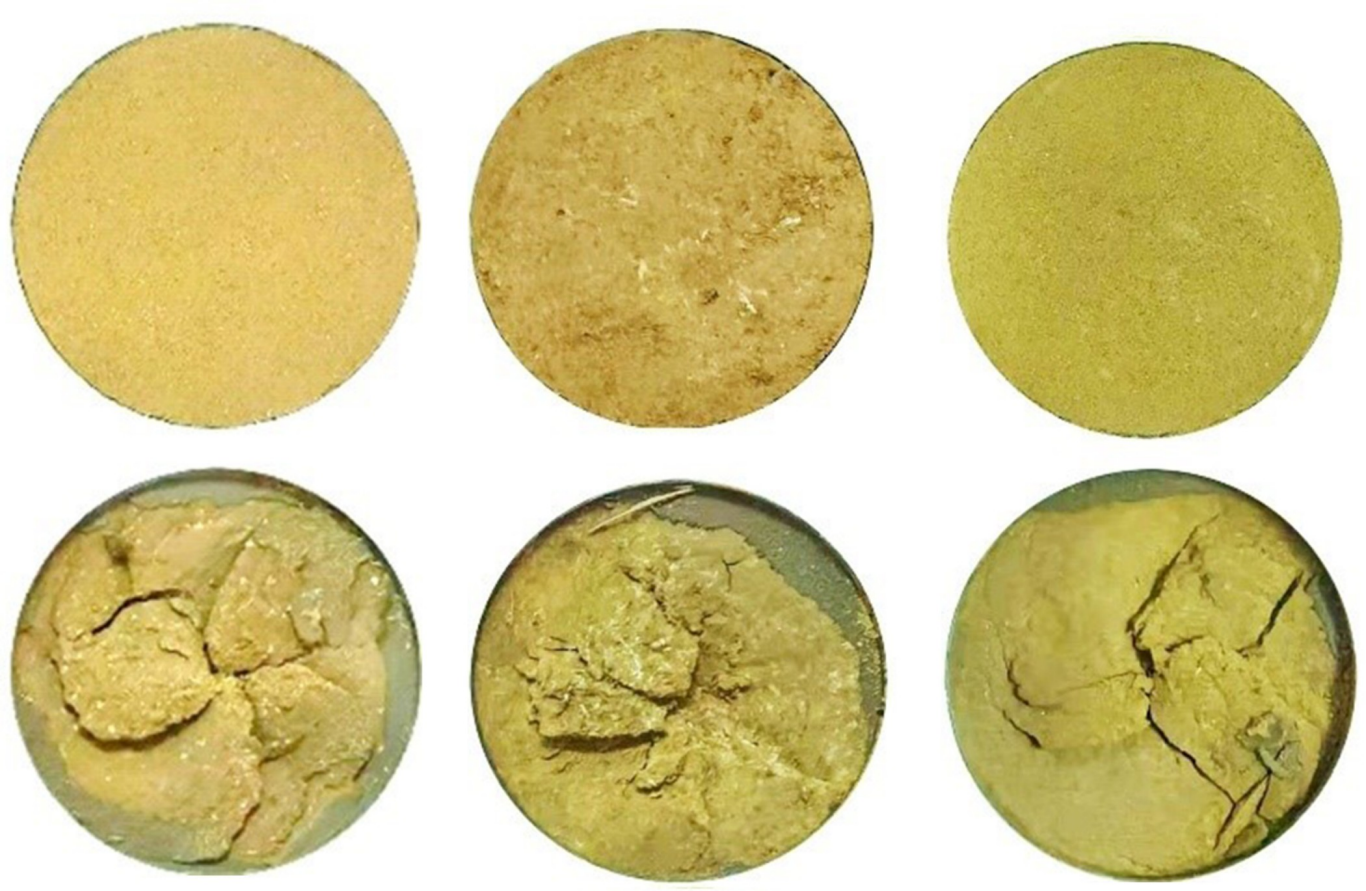
Comparison of the shear surface morphology of 22^#^ - 24^#^ remolded loess specimen before and after the test. **(A**) 22^#^ *w* = 16%. **(B)** 23^#^ *w* = 19%. **(C)** 24^#^ *w* = 22%.

**Fig 11:**
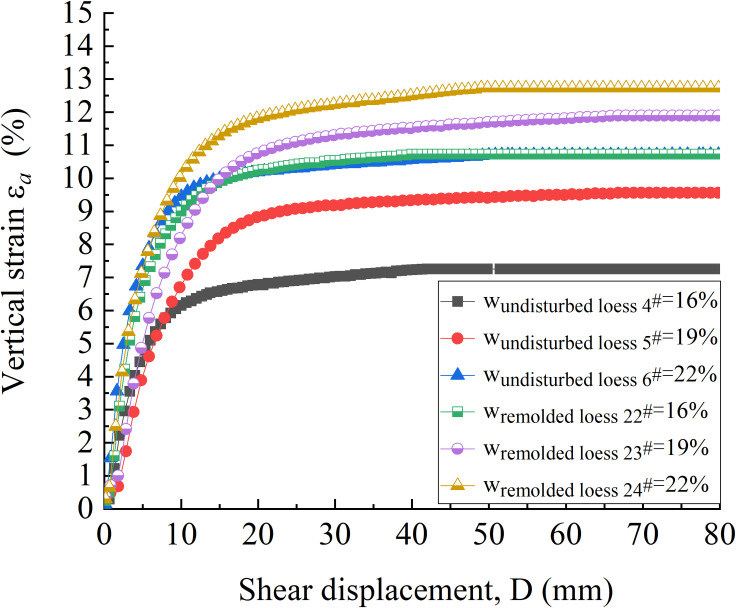
Vertical strain-shear displacement curve of 4^#^ - 6^#^ undisturbed and 22^#^ - 24^#^ remolded loess specimens under different water content.

The undisturbed 4^#^ - 6^#^ and remolded 22^#^ - 24^#^ specimens are used to study the residual strength variation characteristics of undisturbed and remolded loess specimens under different water contents. The shear surface morphology of 4^#^ - 6^#^ and 22^#^ - 24^#^ specimens before and after shear is shown in Figs 9A–9C and 10A–10C, respectively. It can be seen that the shear surface morphology of undisturbed and remolded loess specimens shows very obvious changes before and after shear under three kinds of water content. The shear area of undisturbed 5^#^ - 6^#^ and remolded 23^#^ - 24^#^ specimens is larger than that of 4^#^ and 22^#^ specimens, and the shear depth is deeper. That is, with the increase of water content, the effect of shear failure of loess specimens is more obvious. Fig 11 shows the variation curves of the vertical strain of 4^#^ - 6^#^ and 22^#^ - 24^#^ specimens with shear displacement. It can be seen from Fig 11 that when the shear displacement is 60 mm, the corresponding vertical strains of 4^#^ - 6^#^ specimen are roughly 7.2%, 9.4% and 10.7%; the corresponding vertical strain of the 22^#^ - 24^#^ specimen is roughly 10.7%, 11.7% and 12.7%, respectively. The larger the water content, the larger the vertical settlement of the loess specimen when reaching the residual strength.

**Fig 12:**
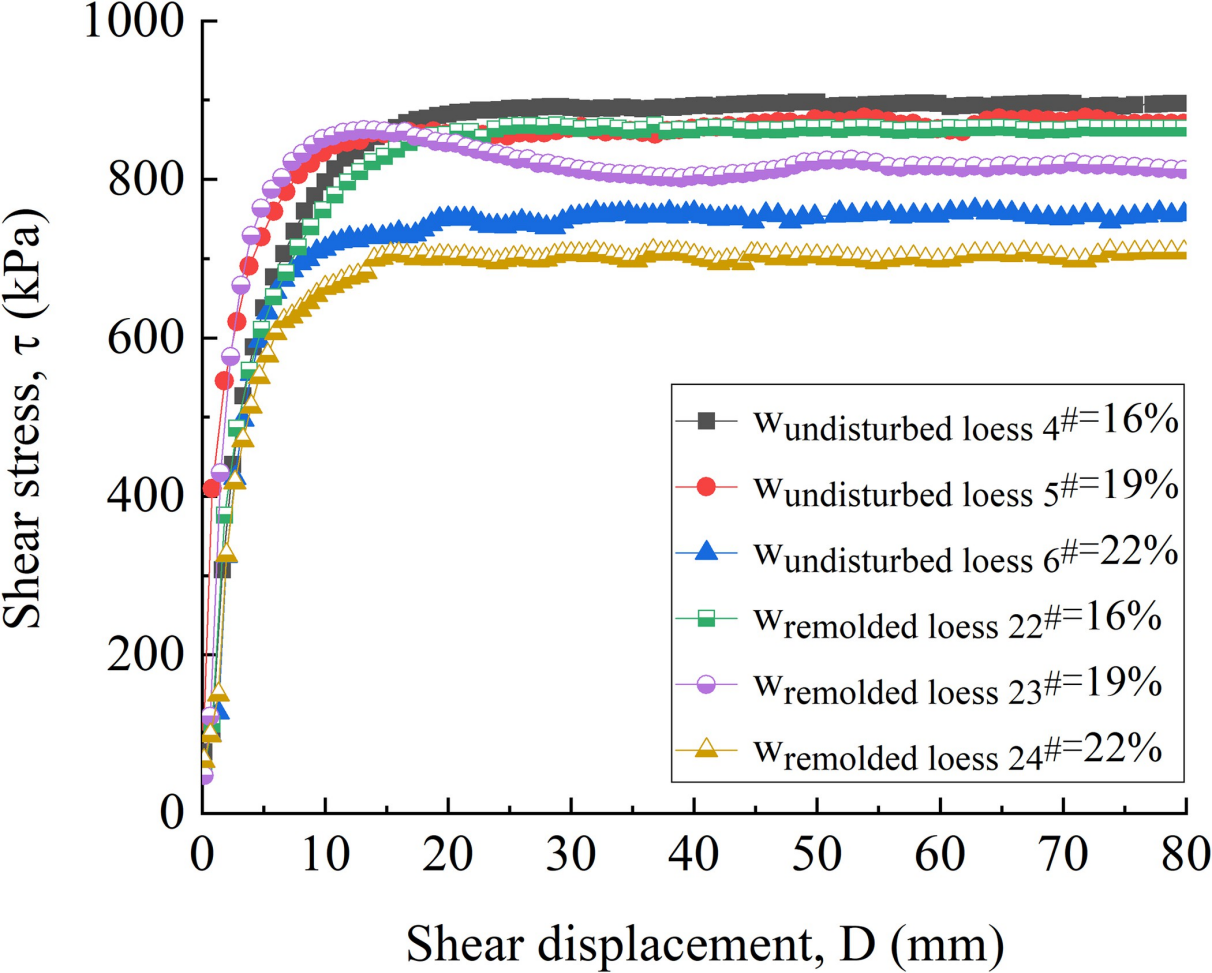
Shear stress-shear displacement curve of 4^#^ - 6^#^ undisturbed and 22^#^ - 24^#^ remolded loess specimens under different water content.

Fig 12 is the shear stress-shear displacement curve of undisturbed 4^#^ - 6^#^ and remolded 22^#^ - 24^#^ specimens. It can be seen from Fig 12 that under the same vertical pressure, the shear stress-shear displacement curves of low water content 4^#^ and 22^#^ specimens are hardening type. When the specimen with higher water content reaches the peak strength, the corresponding shear deformation is small, and the strength softening is also easy to occur (the softening phenomenon of 23^#^ specimen with the water content of 19% is obvious), that is, the process of attenuation from the peak strength includes two stages: the first stage is that the shear dilatancy increases the pore water content and then leads to strain softening, and the second stage is that with the increase of shear strain, the loess particles gradually reach the residual strength along the shear direction [5]. When the shear displacement reaches about 60 mm, the shear stress of each water content specimen has basically reached a stable value, and the specimens have reached the residual strength. Due to the low water content and dry interior of 4^#^ and 22^#^ specimens, the change of pore water transport is not obvious during the shear process, so the shear stress does not decrease significantly with the increase of shear strain. When the water content increases, the strain softening occurs, and the shear stress decreases significantly with the migration of pore water and the rearrangement of particles.

**Fig 13:**
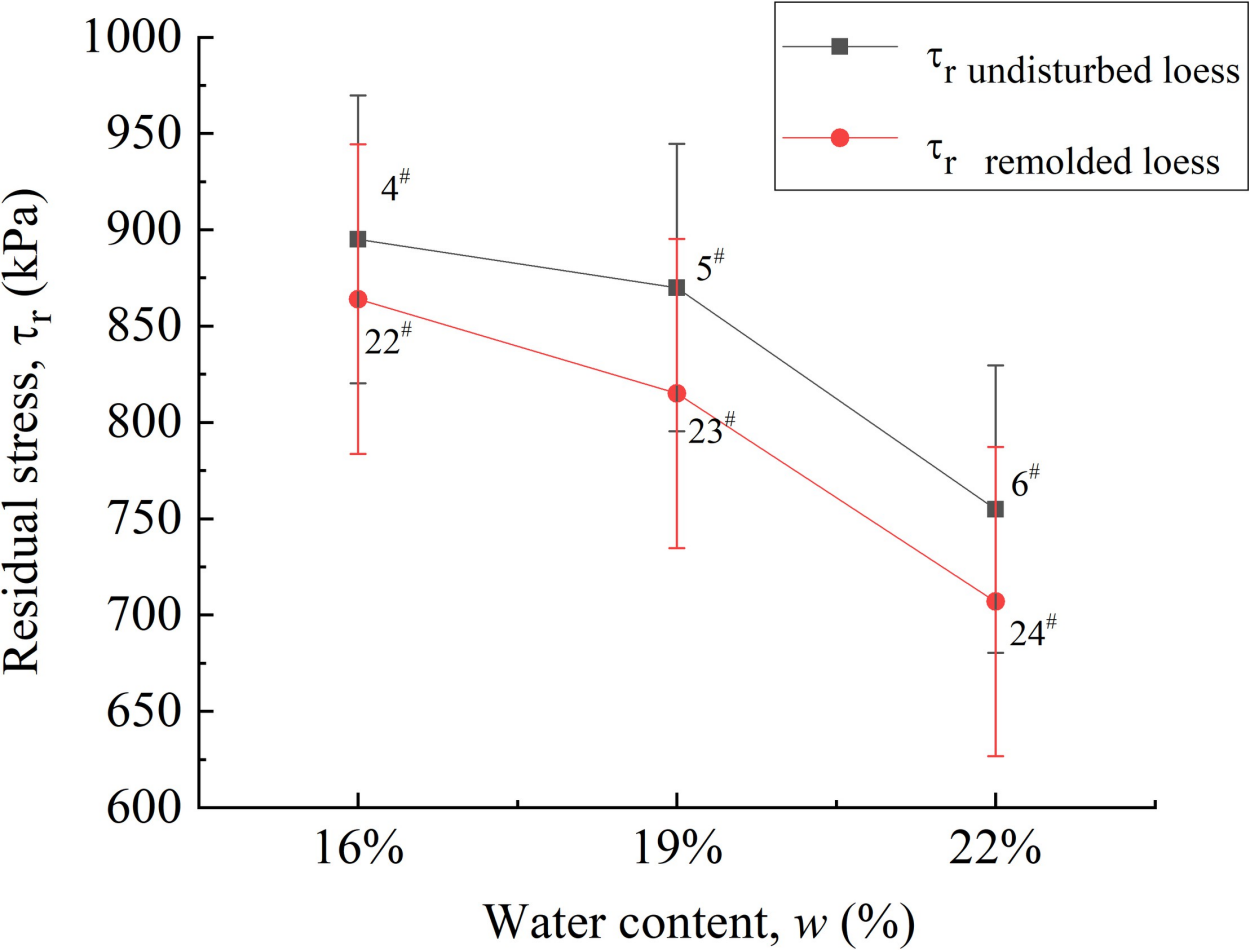
Residual strain-water content curve of 4^#^ - 6^#^ undisturbed and 22^#^ - 24^#^ remolded loess specimens.

It can be seen from the relationship curve between residual strength and water content, as shown in Fig 13 that water content also has a certain influence on residual strength, that is, with the increase of water content, residual strength gradually decreases, and the residual strength of undisturbed loess specimens under the same water content is generally greater than that of remolded loess specimens. Under 16% water content, the residual strength of 4^#^ and 22^#^ loess specimens is 895 kPa and 864 kPa, respectively, and the difference percentage is 3.6%; the residual strength of 5^#^ and 23^#^ loess specimens under 19% water content is 870 kPa and 815 kPa, respectively, and the difference percentage is 6.7%; the residual strength of 6^#^ and 24^#^ loess specimens under 22% water content is 755 kPa and 707 kPa, respectively, and the difference percentage is 6.8%.

### Impact of different vertical pressure on deformation and strength characteristics of undisturbed and remolded loess specimens

**Fig 14:**
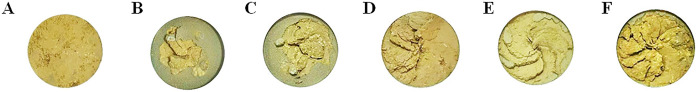
Comparison of the shear surface morphology of 4^#^、7^#^ - 10^#^ undisturbed loess specimen before and after the test shearing (*w* = 16%). **(A)** Before specimen. **(B)** 7^#^ *σ* = 100 kPa. **(C)** 8^#^ *σ* = 200 kPa. **(D)** 9^#^ *σ* = 400 kPa. **(E)** 4^#^ *σ* = 800 kPa. **(F)** 10^#^ *σ* = 1200 kPa.

**Fig 15:**
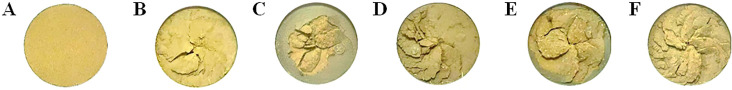
Comparison of the shear surface morphology of 22^#^、25^#^ - 28^#^ remolded loess specimen before and after the test shearing (*w* = 16%). **(A)** Before specimen. **(B)** 25^#^ *σ* = 100 kPa. **(C)** 26^#^ *σ* = 200 kPa. **(D)** 27^#^ *σ* = 400 kPa. **(E)** 22^#^ *σ* = 800 kPa. **(F)** 28^#^ *σ* = 1200 kPa.

The undisturbed 4^#^ - 18^#^ and remolded 22^#^ - 36^#^ specimens are used to study the residual strength variation characteristics of undisturbed and remolded loess specimens under different vertical pressures, and the shear tests under three groups of water contents (16%, 19% and 22%) are performed in parallel. The loess specimens are consolidated and sheared under the vertical pressure of 100 kPa, 200 kPa, 400 kPa, 800 kPa and 1200 kPa, respectively. The shear surface morphology of undisturbed 4^#^, 7^#^-10^#^ and remolded 22^#^, 25^#^ - 28^#^ specimens (water content 16%) before and after shearing are shown in Figs 14A–14F and 15A–15F, respectively. It can be seen that the shear surface morphology of undisturbed and remolded loess specimens before and after shear shows obvious changes under five different vertical pressures. Under the same vertical pressure, the shear band morphology of the two specimens is similar. Among them, the shear failure effect of undisturbed 4^#^, 10^#^ and remolded 22^#^, 28^#^specimens is more obvious than that of undisturbed 7^#^ - 9^#^ and remolded 25^#^ - 27^#^ specimens, that is, with the increase of vertical pressure, the shear failure degree of specimens increases gradually. This is because that the smaller vertical pressure makes the steel interface rotate only on the surface of loess, and the greater the vertical pressure, the better the contact between the steel interface and loess, leading to the failure of deep loess.

**Fig 16:**
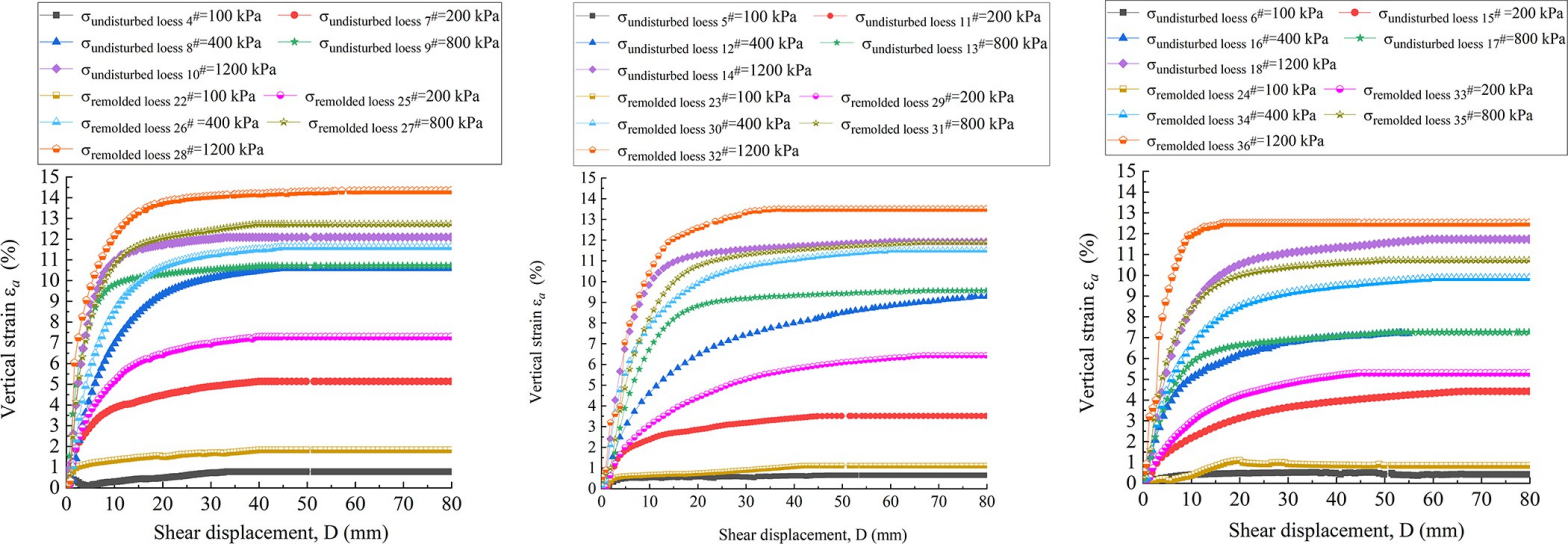
Vertical strain-shear displacement curve of 4^#^ - 18^#^ undisturbed and 22^#^ - 36^#^ remolded loess specimens under different vertical pressure. **(A)** Vertical strain-shear displacement curve of 4^#^、7^#^ - 10^#^ undisturbed and 22^#^、25^#^ - 28^#^ remolded loess specimens (*w* = 16%). **(B)** Vertical strain-shear displacement curve of 5^#^、11^#^ - 14^#^ undisturbed and 23^#^、29^#^ - 32^#^ remolded loess specimens (*w* = 19%). **(C)** Vertical strain-shear displacement curve of 6^#^、15^#^ - 18^#^ undisturbed and 24^#^、33^#^ - 36^#^ remolded loess specimens (*w* = 22%).

**Fig 17:**
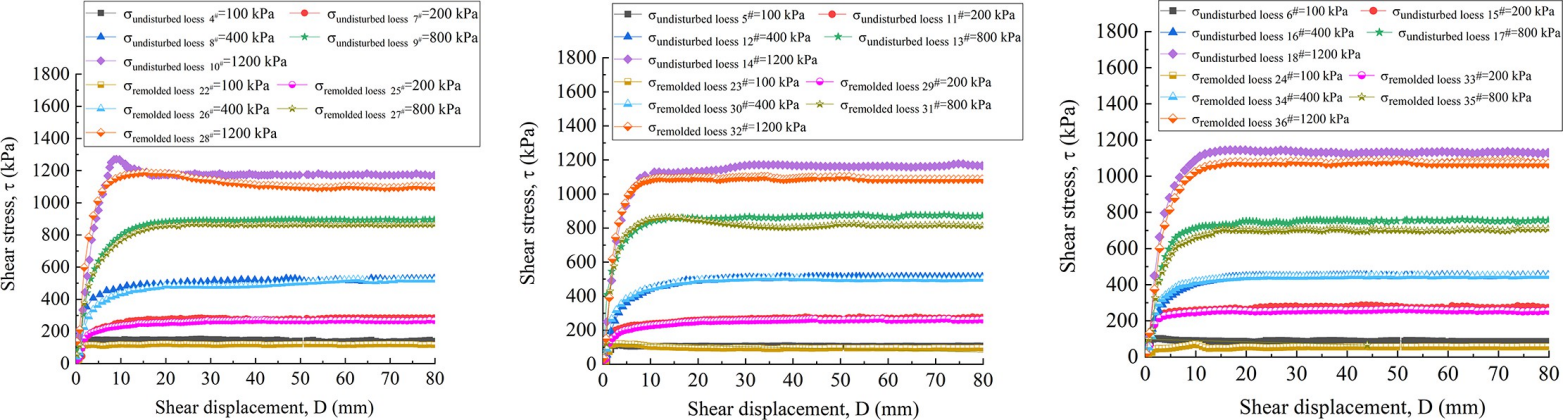
Shear stress-shear displacement curve of 4^#^-18^#^ undisturbed and 22^#^ - 36^#^ remolded loess specimens under different vertical pressure. **(A)** Shear stress-shear displacement curve of 4^#^、7^#^ - 10^#^ undisturbed and 22^#^、25^#^-28^#^ remolded loess specimens (*w* = 16%). **(B)** Shear stress-shear displacement curve of 5^#^、11^#^ - 14^#^ undisturbed and 23^#^、29^#^-32^#^ remolded loess specimens (*w* = 19%). **(C)** Shear stress-shear displacement curve of 6^#^、15^#^ - 18^#^ undisturbed and 24^#^、33^#^-36^#^ remolded loess specimens (*w* = 22%).

Fig 16A–16C are the vertical strain-shear displacement curves of undisturbed and remolded loess specimens with the water content of 16%, 19% and 22%, respectively. It can be seen from Fig 17 that the shear failure of the specimen is basically a shear shrinkage process, and the change trend of the undisturbed and remolded specimens under the same water content and the same vertical pressure is highly consistent. The undisturbed 5^#^, 11^#^ - 14^#^and remolded 23^#^, 28^#^ - 32^#^ specimens with the water content of 19% are analyzed. Under the vertical pressure of 100 kPa, when the shear displacement of the undisturbed 11^#^ and the remolded 29^#^ specimens is 60 mm, the corresponding vertical strains are roughly 0.65% and 1.11%; under the vertical pressure of 200 kPa, when the shear displacement is 60 mm, the corresponding vertical strains of the undisturbed 12^#^ and remolded 30^#^ specimens are roughly 3.51% and 6.44%; under the vertical pressure of 400 kPa, when the shear displacement is 60 mm, the corresponding vertical strains of the undisturbed 13^#^ and remolded 31^#^ specimens are approximately 9.27% and 11.51%, respectively. Under the vertical pressure of 800 kPa, the corresponding vertical strains of the undisturbed 5^#^ and remolded 23^#^ specimens are approximately 9.56%and 11.89% when the shear displacement is 60 mm, respectively. Under the vertical pressure of 1200 kPa, when the shear displacement is 60 mm, the corresponding vertical strains of the undisturbed 14^#^ and remolded 32^#^ specimens are roughly 11.93% and 13.51%. The comparison shows that the vertical strain of undisturbed and remolded loess specimens increases with the increase of vertical pressure. Secondly, under the same vertical pressure, the vertical strain of remolded loess is generally larger than that of undisturbed loess.

Fig 17A–17C are shear stress-shear displacement curves of undisturbed and remolded loess specimens with the water content of 16%, 19% and 22%, respectively. It can be found from the figure that under the same water content, the residual strength of the specimen increases with the increase of vertical pressure, and the slope of the initial stage of the curve also increases with the increase of vertical pressure, indicating that the corresponding shear stress growth rate is larger. For the specimen with water content of 16%, as shown in Fig 17A, during the shear process under the vertical pressure of 1200 kPa, the stress of the undisturbed loess 10^#^ specimen decreases significantly after reaching the peak strength, namely, from 1270 kPa to 1190 kPa, and then the small amplitude fluctuation reaches the stable residual strength of 1170 kPa; after reaching the peak strength, the stress of remolded loess 28^#^ decreases slowly, that is, from 1187 kPa to 1122 kPa, and then the small amplitude fluctuation reaches the stable residual strength 1100 kPa. For the specimens with the water content of 19%, as shown in Fig 17B, when the vertical pressure is 100 kPa and 800 kPa, it can be observed that the stress of the remolded loess 29^#^ and 23^#^ specimens decrease relatively slowly after reaching the peak strength, that is, from 120 kPa to 104 kPa, and from 862 kPa to 840 kPa, respectively. Subsequently, the small amplitude fluctuation gradually reaches the stable residual strength of 95 kPa and 815 kPa, respectively. For the undisturbed 6^#^, 15^#^-18^#^ and remodeled 24^#^, 33^#^ - 36^#^specimens with the water content of 22%, as shown in Fig 17C, the strength hardening is mainly dominated. The shear stress of the specimens under different vertical pressures increases rapidly to a larger strength within a small shear displacement range (0 ~ 10 mm), and then fluctuates slightly within a certain range with the increase of shear displacement, and finally stabilizes at the residual strength.

**Fig 18:**
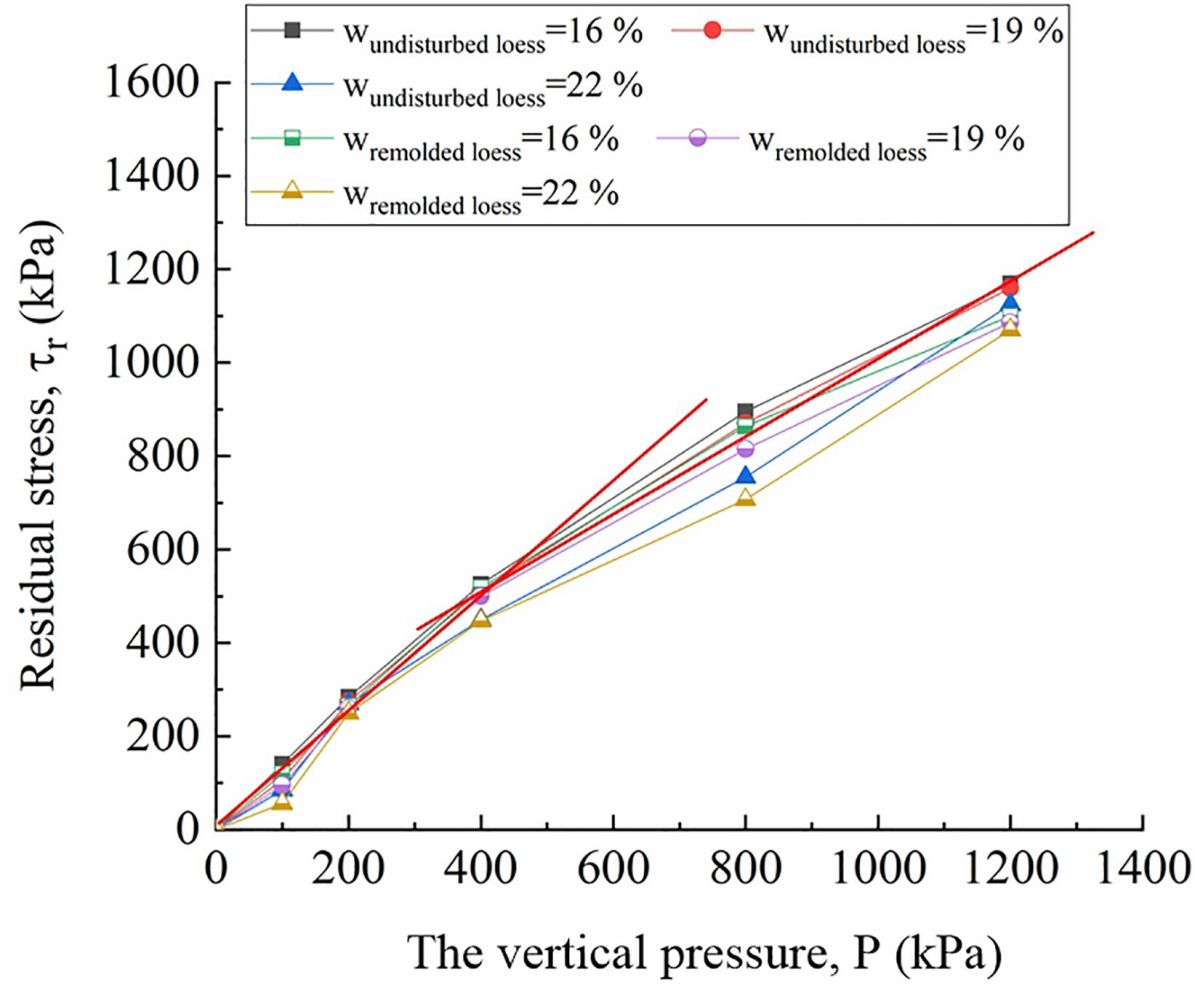
Residual strain- vertical pressure curve of 4^#^ - 18^#^ undisturbed and 22^#^ - 36^#^ remolded loess specimens.

Fig 18 is the residual strength-vertical pressure curve of undisturbed and remolded loess specimens with the water content of 16%, 19% and 22%. The curve in the fitting diagram shows that between the residual strength and vertical pressure of undisturbed and remolded loess specimens under the same water content, the curve is divided into two parts, i.e., low-pressure area and high-pressure area with the vertical pressure of 400 kPa as the boundary, showing a strong linear relationship. The change trend of residual strength conforms to the Mohr-Coulomb Law:

$$\tau_{r}=c_{r}+\sigma_{n}tan\varphi_{r}$$ (5)

where *τ*_*r*_ refers to the residual strength (kPa) when the specimen reaches stability; *σ*_*n*_ represents the current vertical pressure on the specimen (kPa); *φ*_*r*_ stands for the residual internal friction angle when the specimen reaches stability (the value is related to the interface material, which is the internal friction angle of the steel-loess interface); *c*_*r*_ is the residual cohesive force when the specimen reaches stability (i.e., the cohesive force of the loess itself); *c*_*r*_ and *tanφ*_*r*_ are the intercept and slope of the straight line, respectively, which are determined by using the least square method.

The residual strength index of unsaturated loess plays an important role in the stability analysis of loess interface contact with other interfaces, therefore, it is necessary to analyze the residual strength index. The residual strength indexes of undisturbed and remolded loess specimens under the same water content are obtained from Formula (5), as shown in Table 3.

**Table 3:** Residual strength index of 4^#^-18^#^ undisturbed and 22^#^ - 36^#^ remolded loess specimens.

*ρ*_*d*_ (g/cm3)	*v*/(mm/min)	*w* (%)	Specimen Nos.	Residual strength index			
				*c*/(kPa)		*φ*/(°)	
				<400 kPa	≥400 kPa	<400 kPa	≥400 kPa
1.35	1.0	16%	undisturbed loess (4^#^, 7^#^-10^#^)	30.2	33.7	41.8	44.1
			remolded loess (22^#^, 25^#^-28^#^)	24.3	25.5	40.2	42.3
		19%	undisturbed loess (5^#^, 11^#^-14^#^)	25.6	28.5	41.4	43.8
			remolded loess (23^#^、29^#^-32^#^)	20.2	21.3	39.8	42.0
		22%	undisturbed loess (6^#^、15^#^-18^#^)	20.6	23.2	40.9	43.3
			remolded loess (24^#^、33^#^-36^#^)	16.1	17.7	39.5	41.7

## Discussion

A large number of previous studies have focused on the variation of shear strength of remolded loess under large shear displacement at different shear rates, water contents and vertical pressures. However, the comparison of interfacial shear tests of undisturbed and remolded loess under the influence of related factors is still rare. In the following, the influence mechanism of large deformation shear behavior of undisturbed and remolded loess and the characteristics of strength difference between them are discussed to further reveal the influence of structure on the mechanical properties of loess interface.

### Impact of microstructure on interfacial shear strength

**Fig 19:**
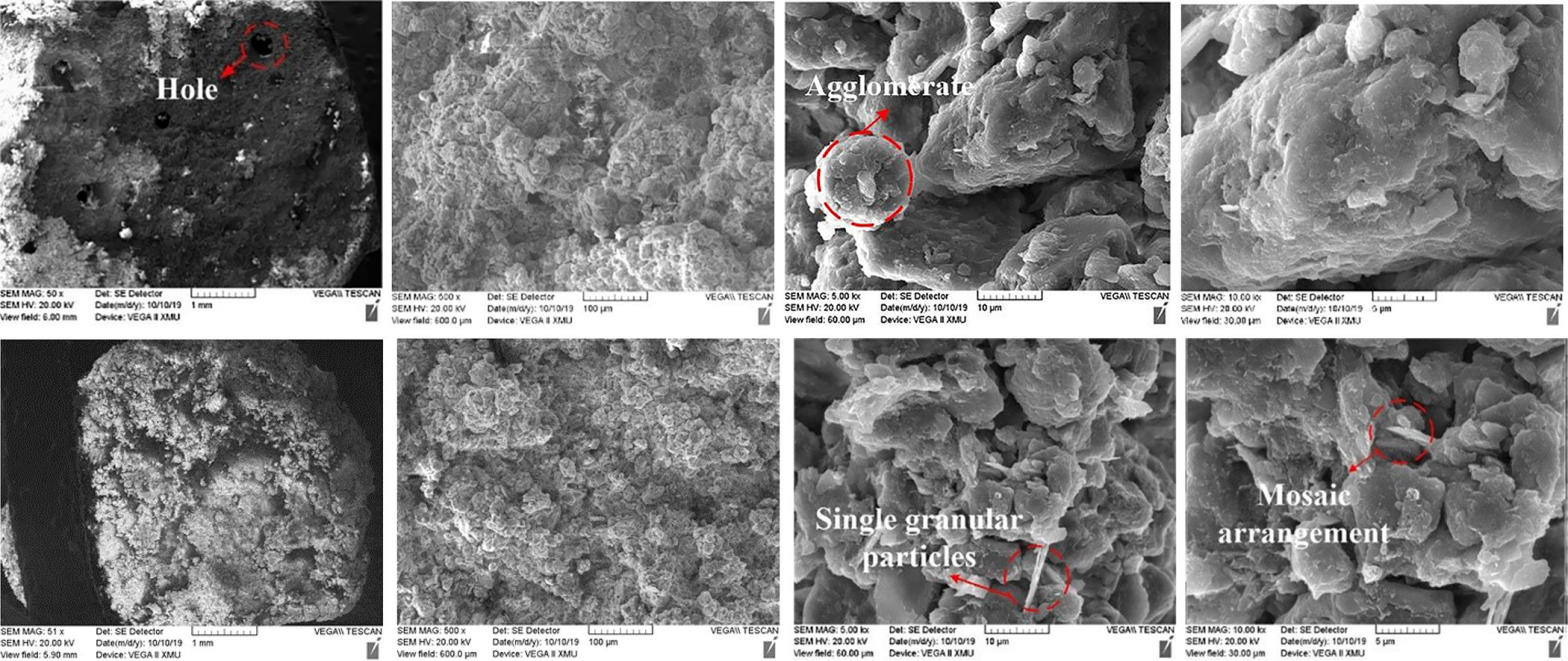
SEM image of undisturbed & remolded loess.

**Fig 20:**
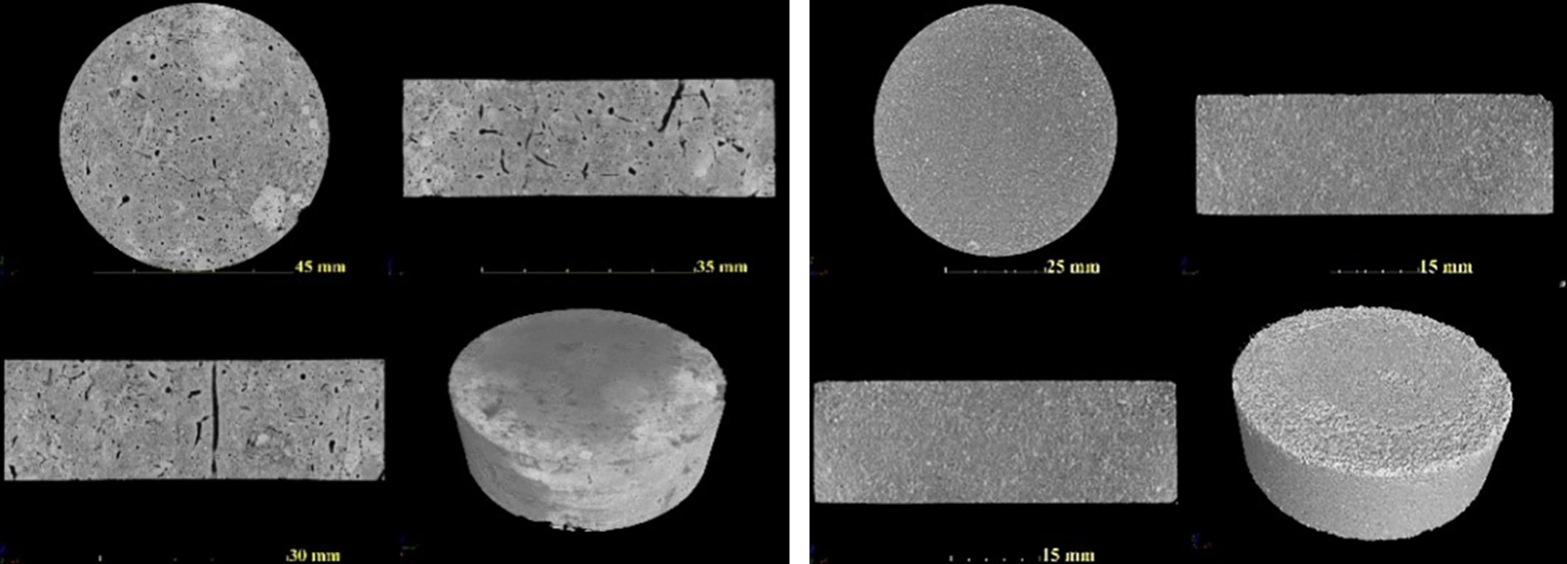
Three views and three-dimensional reconstruction of the ring cutter specimen. **(A)** Undisturbed loess. **(B)** Remolded loess.

Scanning electron microscopy was used to observe the microstructure of undisturbed and remolded loess specimens. The final imaging results at different magnifications (50, 500, 5000, and 10000 times) are shown in Fig 19. At the magnification of 500–5000, a very clear loess particle morphology can be observed, in which the skeleton particles of undisturbed loess show obvious edges and corners; the microstructure of remolded loess is destroyed due to disturbance, therefore, the angular shape of skeleton particles is close to round. When the magnification is 10000, the arrangement and contact relationship of loess particles can be observed. The arrangement of skeleton particles of undisturbed loess is mainly overhead arrangement, and the contact relationship is mainly point contact, surface contact and cementation contact. The skeleton particles of remolded loess are mainly inlaid arrangement, and the contact relationship is mostly point contact and surface contact. CT 3D scanning of the original and remolded ring knife specimens (diameter of about 20 mm and height of about 61.8 mm). The three-view (overlooking, left-view and main-view) and three-dimensional reconstruction of the specimen are shown in Fig 20A and 20B. It can be seen that the undisturbed loess shows obvious cracks and pores, and the number of large pores is relatively large. The remolded loess specimens are dense without obvious cracks and large pores [53].

For undisturbed and remolded loess specimens, the shear strength of loess-steel interface mainly comes from the friction between steel interface and loess specimen particles and the friction between loess specimen particles. When the steel friction plate rotates and shears, the loess particles bite each other, and the force chain formed transfers the external load. Due to the obvious edge angle of undisturbed loess particles, the friction between particles and that between steel interface and particles are larger. However, the shape of disturbed particles of remolded loess is close to muddy circle, therefore, the interlocking friction between particles is small, and the sliding effect between steel interface and itself increases. In addition, the cement between the undisturbed loess particles provides the particles with strong cementation, and the cementation contact in the remolded loess is destroyed. Therefore, the structure of the undisturbed loess specimen is relatively stable, which can better resist the external force damage. The connection of the remolded loess specimen particles is not stable, and the resistance is poor. The difference in strength between the two is mainly reflected by the structural difference.

### Impact law of shear rate on mechanical properties of interface

The initial state of loess specimen after consolidation has certain structure. During the shearing process, the initial structure of the specimen is destroyed, and the shear surface is formed under the combined action of the effective vertical stress and the torsion of the shear box. The particles on the shear surface are redistributed after shearing. The occlusion and friction between loess particles gradually decrease with the increase of shear displacement, and the shear strength of loess specimens gradually tends to be stable.

Combined with Figs 6 and 7, it can be seen that at the beginning of the shear displacement (0 ~ 10 mm), the shear stress of the specimen increases most rapidly, and the vertical strain is also in the fastest growing stage. Subsequently, the vertical strain increases slowly with the increase of the shear displacement, and the shear stress also changes slowly within a certain displacement range, and finally reaches a stable residual strength, indicating that there is a certain influence relationship between the change of specimen height and the shear stress. It can also be seen from Fig 7 that the vertical strain of the undisturbed and remolded specimens under different shear rates is lower than that under the influence of other factors. Therefore, it can be considered that the height of the specimen is basically the same when it reaches the residual strength. Different shear rates may affect the dissipation rate of pore water and the degree of directional arrangement of particles on the shear surface gradually formed by loess specimens, therefore, the shear stress at the initial stage of shear is slightly different. The larger the shear rate near the shear surface, the more intense the disturbance. The formation and development of the shear zone will result in a sharp increase in pore water pressure, and the shear stress will increase. Therefore, the peak strength increases with the increase of shear rate. This conclusion is similar to many research conclusions [50–52], but the influence of shear rate on residual strength is complex, and there is no uniform conclusion.

Under a certain vertical pressure, the lower the shear rate, the longer the time given to the redistribution and arrangement of loess particles, and the better the occlusion and embedding effect between the loess particles on the loess surface. The contact between the friction plate and the loess particles on the loess surface is sufficient. At this time, the bonding effect between the friction plate and the remolded loess and the friction force cannot resist the shear effect of the friction plate torque, which makes the steel-loess interface slip and the loess surface become smooth and dense. At relatively high shear rate, sliding friction occurs between loess particles and steel interface, which rapidly increases the interfacial shear stress and results in the “bonding” effect between steel interface and loess particles. However, due to the insufficient adjustment position of loess particles and the time of mutual occlusion and immobilization, the cohesion formed between steel interface and loess particles exceeds that between loess particles, which promotes the formation of shear surface, and produces groove and flake shear products. In addition, when the shear rate is slow, the ’bonding’ effect between the steel interface and the loess particles is not strong, but the “false cohesion” caused by the occlusion between the concave and convex particles on the metal friction plate is equivalent to the adsorption of the metal friction plate on the loess particles. When the shear rate is relatively high, the “bonding” effect between the steel interface and the loess particles is enhanced, which destroys the internal bonding of the specimen, and results in the local deep sliding failure of the specimen. The residual cohesion is close to the cohesion of the loess itself, rather than the “pseudo cohesion” between the friction plate and the clay particles.

Under a certain vertical pressure, the larger the shear rate, the smaller the vertical strain. The shear stress is mainly affected by the shear rate before reaching the ultimate stable residual strength. Therefore, at a large shear rate, due to the insufficient space for reorganization of loess particles, the speed to reach the alignment direction is low. When the shear displacement reaches the residual strength, the height of the specimen does not change significantly. At small shear rate, the loess specimen is gradually damaged, therefore, the sliding reorganization of loess particles has a complete buffer adjustment space, and the vertical deformation is more obvious.

**Fig 21:**
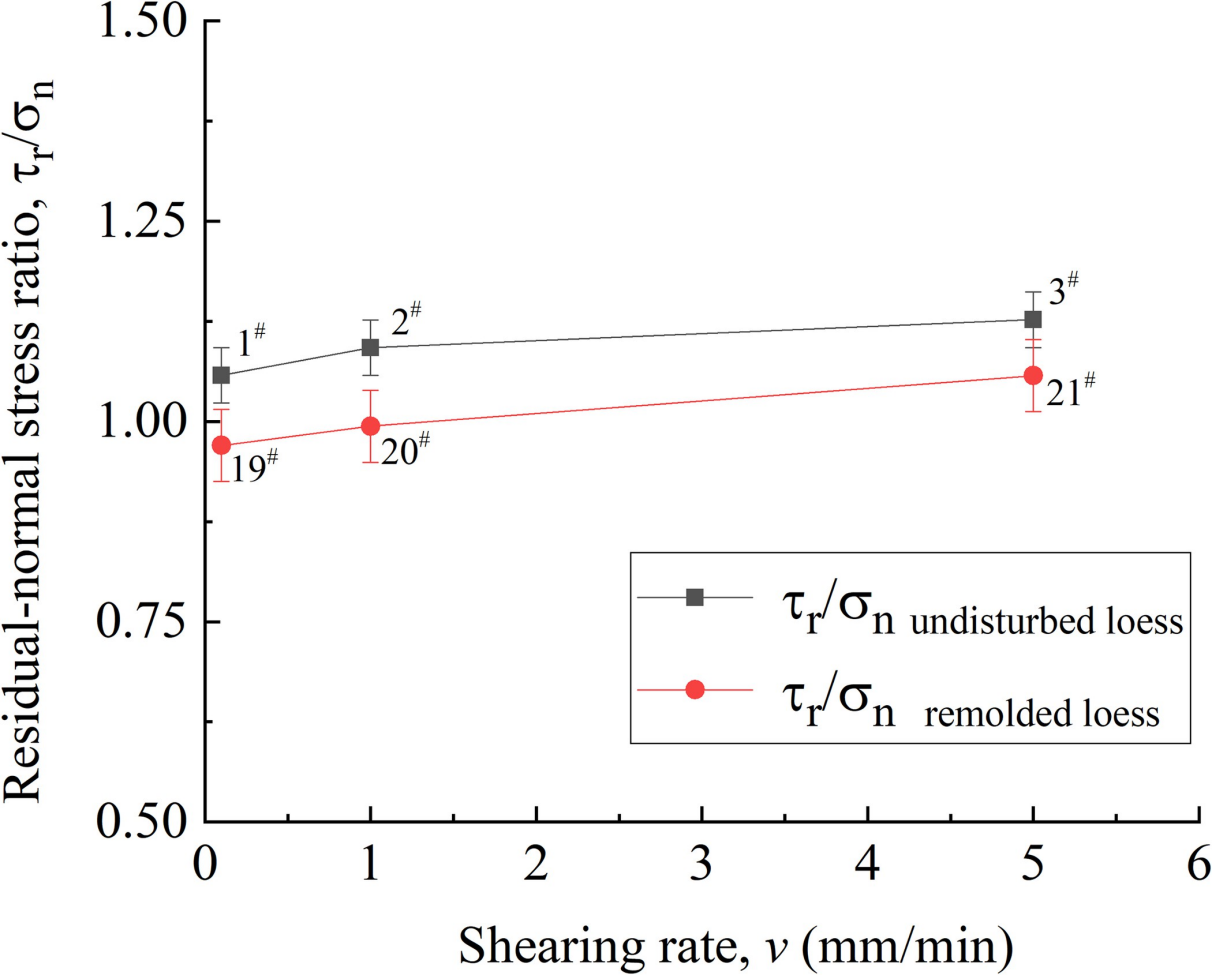
Residual friction coefficient-shear rate curve of 1^#^ - 3^#^ undisturbed and 19^#^ - 21^#^ remolded loess specimens.

Lupini [54] et al. proposed that the residual strength can be reflected by the friction coefficient under different vertical pressures. Because the shear rate of 0.01 ~ 100 mm / min is more suitable for the loess sliding state in actual engineering construction, it can be seen from Fig 21 that when the shear rate is 0.1 mm / min, 1.0 mm / min and 5.0 mm / min, the residual friction coefficients of undisturbed loess specimens are 1.06, 1.10 and 1.13, respectively, and the difference range is 2.0% ~ 6.6%. The residual friction coefficients of remolded loess specimens are 0.97, 0.99 and 1.06, respectively, and the difference range is 2.0% ~ 9.3%. At three different shear rates, for undisturbed and remolded loess specimens, their residual friction angles can be approximately considered as a constant, indicating that the shear rate has little effect on the residual friction strength of loess specimens [55–57].

This is because that in the later stage, with the increase of shear displacement, the pore water on the shear surface dissipates and the particles are reorientation is completed [58]. At this time, the specimen is with residual friction strength. The strength under this state is mainly related to the shear stress state, and the shear rate has little effect on it. In addition, because the degree of particle reorientation of undisturbed loess specimen is higher than that of remolded loess specimen, the residual friction angle is slightly larger than that of remolded loess specimen, therefore, the shear strength of undisturbed loess specimen is slightly higher than that of remolded loess specimen.

### Impact law of water content on mechanical properties of interface

**Fig 22:**
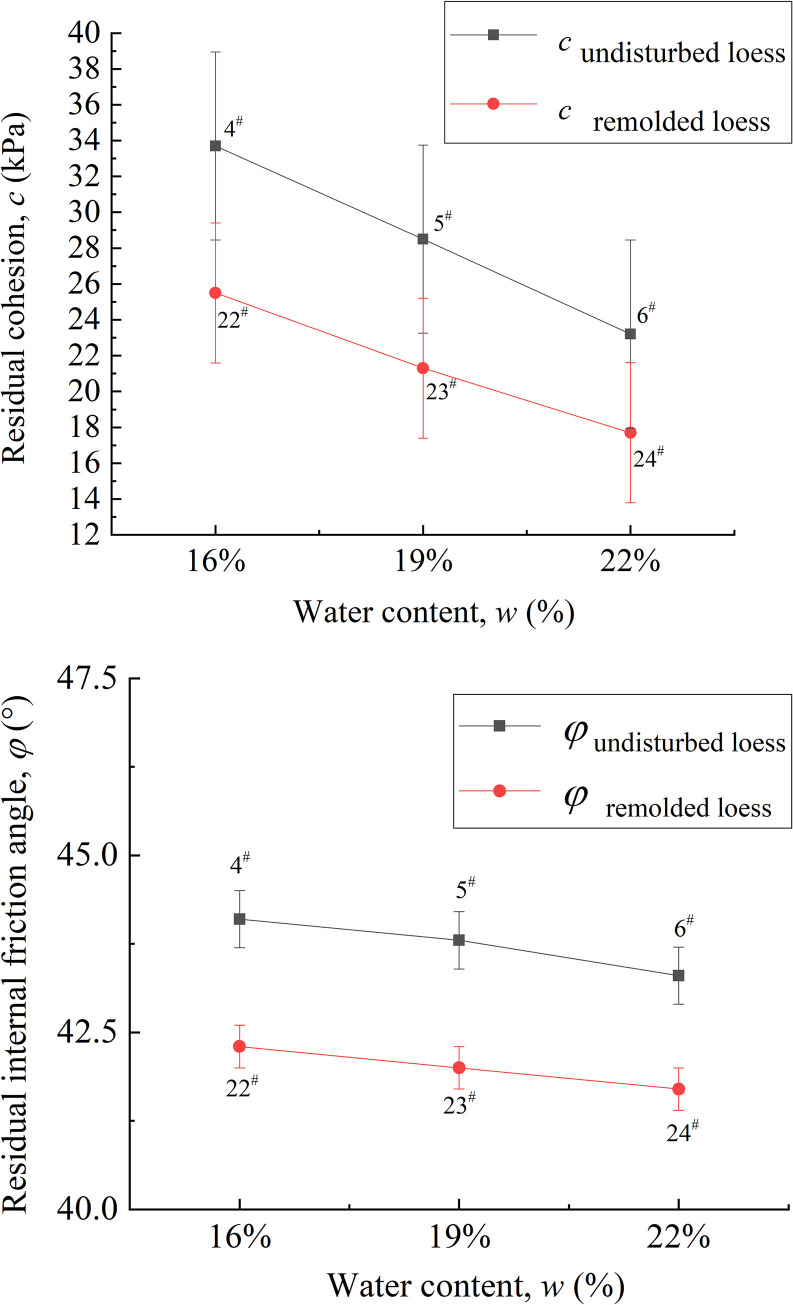
Residual cohesion & residual friction coefficient- water content curve of 4^#^ - 6^#^ undisturbed and 22^#^ - 24^#^ remolded loess specimens. **(A)** Residual cohesion- water content curve. **(B)** Residual friction coefficient- water content curve.

The residual cohesive strength parameters mainly include residual cohesive force and residual internal friction angle. According to Table 3, the relationship between water content and residual cohesive force and residual internal friction angle can be obtained, as shown in Fig 22A and 22B. It can be seen that with the increase of water content, the cohesive force and internal friction angle corresponding to the residual strength of loess specimens decrease, which is due to the decomposition of soluble minerals inside the loess specimen, resulting in the decrease of cohesive force between loess particles. In addition, due to the lubrication effect between loess particles, the friction between particles also decreases with the increase of water content [59, 60]. The residual cohesion of 4^#^ - 6^#^ undisturbed specimen is 33.7, 28.5 and 23.2, respectively, and the difference range is 18.25% ~ 45.26%. The residual cohesion of 22^#^ - 24^#^ remolded specimen is 25.5, 21.3 and 17.7, respectively, and the difference range is 19.72% ~ 44.07%. The residual internal friction angles of the undisturbed 4^#^-6^#^ specimen are 44.1, 43.8 and 43.3, respectively, and the difference range is 0.68% ~ 1.85%. The residual internal friction angles of the remolded 22^#^ - 24^#^ specimen are 42.3, 42 and 41.7, respectively, and the difference range is 0.71% ~ 1.44%. It can be seen that the variation range of residual cohesive force is much larger than that of residual internal friction angle, therefore, the residual internal friction angle is the dominant factor to support the residual strength.

With the increase of water content, the thickness of water film around the particles in the loess increases, the ability of the specimen to resist the torsional shear failure of the friction plate on the shear head decreases, the degree of sliding and slip in the annular shear increases, and the strength of the steel-loess interface decreases. Due to the strong cohesive force between the undisturbed loess specimens in the initial state, the cohesive force of the remolded loess specimen has been destroyed to some extent due to human disturbance in the process of specimen preparation. Therefore, the residual cohesive force of the remolded loess specimens is lower than that of the undisturbed loess specimens under the same water content. Secondly, since the particle morphology of undisturbed loess specimens is mainly composed of fine-grained particles cemented, and it has obvious angularity; the particle morphology of the remolded loess specimen is mainly single particle, which is close to round shape, therefore, the residual internal friction angle between the particles of the undisturbed loess specimen is slightly larger than that of the remolded loess specimen. In this case, the interface residual strength of the undisturbed loess specimen under the same water content is greater than that of the remolded loess specimen.

By comparing the vertical strain changes of loess specimens before and after shearing, it can be seen that the void ratio of loess specimens is changing, therefore, the change of loess structure can be described from this aspect. This is because that there are a large number of pores in the loess specimen, and the larger vertical pressure pushes out the water and gas in the loess specimen, thereby reducing the void ratio. Subsequently, the water content of the loess specimen gradually increases, and the cementing material between the particles in the loess specimen is gradually dissolved by the thickened water film formed around the particles. The bonding strength between the loess particles is weakened, and the void ratio is greatly reduced, resulting in the decrease of compression modulus and the increase of vertical settlement. On the other hand, water content usually affects the aggregate composition of particles in loess. The dry specimen is composed of smaller aggregates of loess particles, while in the wet state, the specimen is mainly composed of larger and aggregates which are more easily deformed [61]. Therefore, when the water content is low, the particles and pores are small, which makes the matrix suction in the pores high, and the bound water close to the surface of the loess particles is subjected to strong electric molecular gravity, showing strong water film bonding force, and the bonding effect is obvious. The stability of the electric double layer structure on the surface of the loess particles is strong, and the loess shows strong cohesion. Under the condition of high water content, the thickness of the combined water film inside the loess increases, the matric suction decreases, the connection between the water films decreases, the bonding effect decreases, and the cementation between the loess particles gradually loses. The influence of water on the strength of the loess changes from cohesion to lubrication, and the relative deformation of the particles is large. The hardness of the particles also gets lower and lower, and the strength of the loess specimen itself decreases, thereby reducing the residual strength of the steel-loess interface.

### Impact law of vertical pressure on mechanical properties of interface

When the water content is constant, the shear strength increases with the increase of vertical pressure for the undisturbed and remolded loess specimens with stable residual strength under different vertical pressures, which is consistent with the conclusion of partial ring shear and interface direct shear test [62–66]. First of all, the loess particles on the shear surface will move and break during the shear process, which may make the particle size and directional arrangement of the loess particles on the shear surface of the specimen before and after shear under various normal stresses different, thus affecting the residual internal friction angle when the residual strength is finally reached. Through analysis, it is found that under different vertical pressures, the influence of the change of loess particle size on the residual internal friction angle on the shear surface before and after the shear of loess specimen can be ignored. Therefore, the directional arrangement degree between particles has a great influence on the residual internal friction angle on the shear surface [62, 67]. It can also be seen from Fig 18 that when the vertical pressure is low (0 ~ 200 kPa), the linearity between the residual strength and the vertical pressure is relatively low, which is consistent with the conclusion of some researches. In another word, in the lower vertical pressure zone, there is a strong nonlinear relationship between the residual strength and the vertical pressure [62, 68], which should be caused by the incomplete orientation of the viscous particles on the shear plane along the shear direction [69]. When the vertical pressure increases to a certain extent (200 ~ 1200 kPa), the linearity between residual strength and vertical pressure increases. Secondly, under the same water content, the residual internal friction angle and residual cohesion in the high-pressure zone are generally larger than those in the low-pressure zone, which should be due to the fact that the directional arrangement of particles on the shear plane is stable, and the larger the vertical pressure, the higher the degree of directional arrangement of particles. In addition, the vertical deformation reflects the change of the void ratio of the loess, that is, with the increase of the vertical pressure, the void ratio decreases when the specimen reaches the residual strength. The smaller the void ratio of the loess, the more contact points between the particles, so that the increase of the internal friction angle of the residue increases the residual strength.

Through the analysis of particle matter, it is found that the arrangement of loess particles in the loess specimen is relatively dense, and multiple paths for transferring external load are formed between adjacent loess particles in different forms of contact, called “force chain”. A number of “force chains” crisscross each other in the loess particle system to form a network, and get through it to transfer the external load and the weight of the particle system itself. Seguin A [70] quantifies the internal pressure of each particle through tests, and emphasizes two kinds of “force chain” networks existing in particle materials, i.e., bearing network (“force chain” strong network) and dissipative network (“force chain” weak network). The “force chain” network can reflect the arrangement relationship of the spatial position of the particles. Among them, the arrangement form of the undisturbed loess skeleton particles is mainly overhead arrangement, and that of the remolded loess skeleton particles is mainly mosaic arrangement. Therefore, under the same vertical pressure, the loess particles on the shear surface of the undisturbed loess re-adjust the arrangement direction earlier, and the stable speed is relatively high, and the arrangement degree is high. In addition, the “force chain” network formed between particles is also different due to different particle surface friction and gradation. In general, the particle surface friction has a great impact on the structure and stability of the “force chain”. It can be seen that because of the relatively large content of coarse particles in undisturbed loess specimens, the larger particles in the loess specimen are broken and filled with pores during the shear process, which makes the contact between particles closer, and further increases the number of strong “force chain”. Due to the obvious edge angle of undisturbed loess particles, the friction force on the particle surface is enhanced, and the “force chain” is more stable. The internal particles of the remolded loess specimen are relatively smooth, which weakens the friction force on the surface of the particles, resulting in poor stability of the “force chain”. Finally, the residual strength of the undisturbed loess specimen is greater than that of the remolded loess specimen under the same water content.

In this paper, the effect of shear rate, water content and vertical pressure on the deformation characteristics and mechanical properties of steel-loess interface residual strength are discussed. However, there are some shortcomings in this test. In the macro aspect, more groups of undisturbed and remolded loess specimens with different initial dry densities under different shear rates and vertical pressures should be controlled. While in the micro aspect, the SEM scanning of the shear surface morphology before and after shearing under the influence of relevant factors should be supplemented. In addition, the shear strength of the interface between the undisturbed and remolded loess specimens under the same initial conditions and the solid surface with different roughness will also be different. Therefore, in the future, the influence of structure on interface strength will be further studied from the above aspects.

## Conclusion

Through the sorting and comparative analysis of the interface shear test data of unsaturated undisturbed and remolded loess under the same physical and mechanical conditions, it is found that different initial conditions have different degrees of influence on the mechanical properties of steel-loess interface and the deformation strength characteristics of residual loess. The mechanism of structural effect on the mechanical deformation characteristics of steel-loess interface is discussed from the structural point of view. The main research results include:

1. For unsaturated undisturbed and remolded specimens with the same water content, dry density and vertical pressure, at the shear rates of 0.1 mm / min, 1.0 mm / min and 5.0 mm / min, the residual friction coefficients of undisturbed loess specimens are 1.06, 1.10 and 1.13, respectively, and the difference range is 2.0% ~ 6.6%; the residual friction coefficients of remolded loess specimens are 0.97, 0.99 and 1.06, respectively, and the difference range is 2.0% ~ 9.3%, therefore, the shear rate in the test range has little effect on the residual strength. The vertical strain of the undisturbed and remolded specimens under different shear rates is lower than that under the influence of other factors. It can be considered that the height of the specimen when reaching the residual strength is basically the same, and the residual strength of the specimen is mainly related to the shear stress state. In addition, because the degree of particle reorientation of undisturbed loess specimens is higher than that of remolded loess specimens, the residual friction angle is slightly larger than that of remolded loess specimens, therefore, the shear strength of undisturbed loess specimens is slightly higher than that of remolded loess specimens.
2. Under different water content of unsaturated undisturbed and remolded specimens, the higher the water content, the smaller the residual cohesion and residual internal friction angle between the specimen particles, and the lower the residual strength. In addition, due to the strong cohesion between particles of undisturbed loess specimens in the initial state, the cohesion of remolded loess specimens has been destroyed to a certain extent due to human disturbance in the specimen preparation process. Therefore, under the same water content, the residual cohesion of remolded loess specimens is lower than that of undisturbed loess specimens. Secondly, since the particle morphology of undisturbed loess specimens is mainly composed of fine-grained particles cemented, and it has obvious angularity; the particle shape of the remolded loess specimen is mainly single particle, which is close to round shape, therefore, the residual internal friction angle between the particles of the undisturbed loess specimen is slightly larger than that of the remolded loess specimen. The residual strength of undisturbed loess specimen is greater than that of remolded loess specimen under the same water content.
3. When the water content is constant, for unsaturated undisturbed and remolded loess specimens under different vertical pressures, the shear strength increases with the increase of effective vertical stress. And under the same water content, the residual strength of undisturbed loess specimen is greater than that of remolded loess specimens. First of all, since the arrangement form of undisturbed loess skeleton particles is mainly overhead arrangement, and the arrangement form of remolded loess skeleton particles is mainly mosaic arrangement, under the same vertical pressure, the loess particles on the shear surface of undisturbed loess readjust the arrangement direction earlier, the speed of reaching stability is relatively fast, and the arrangement degree is high. Besides, the residual internal friction angle is relatively large. Secondly, the intact cement between undisturbed loess particles provides it stronger cohesive force than the remolded loess particles damaged by cement. Therefore, the residual strength of undisturbed loess specimens is generally greater than that of remolded loess specimens under the same vertical pressure.

The research results can further provide a certain experimental basis for revealing the influencing factors of the shear strength of the steel-loess interface and the influence mechanism of the structure on the mechanical properties of the interface, thereby providing a useful reference for the construction of pile foundation, anchor cable, anchor bolt and other projects in the loess area of central and western China.
